# MAP4K3 mediates amino acid-dependent regulation of autophagy via phosphorylation of TFEB

**DOI:** 10.1038/s41467-018-03340-7

**Published:** 2018-03-05

**Authors:** Cynthia L. Hsu, Elian X. Lee, Kara L. Gordon, Edwin A. Paz, Wen-Chuan Shen, Kohta Ohnishi, Jill Meisenhelder, Tony Hunter, Albert R. La Spada

**Affiliations:** 1 Department of Pediatrics, University of California, San Diego, La Jolla, CA 92093 USA; 2 Departments of Neurology, Neurobiology, and Cell Biology, Duke Center for Neurodegeneration and Neurotherapeutics, Duke University School of Medicine, Durham, NC 27710 USA; 3 Molecular and Cellular Biology Laboratory, Salk Institute for Biological Studies, La Jolla, CA 92037 USA

## Abstract

Autophagy is the major cellular pathway by which macromolecules are degraded, and amino acid depletion powerfully activates autophagy. MAP4K3, or germinal-center kinase-like kinase, is required for robust cell growth in response to amino acids, but the basis for MAP4K3 regulation of cellular metabolic disposition remains unknown. Here we identify MAP4K3 as an amino acid-dependent regulator of autophagy through its phosphorylation of transcription factor EB (TFEB), a transcriptional activator of autophagy, and through amino acid starvation-dependent lysosomal localization of MAP4K3. We document that MAP4K3 physically interacts with TFEB and MAP4K3 inhibition is sufficient for TFEB nuclear localization, target gene transactivation, and autophagy, even when mTORC1 is activated. Moreover, MAP4K3 serine 3 phosphorylation of TFEB is required for TFEB interaction with mTORC1-Rag GTPase-Ragulator complex and TFEB cytosolic sequestration. Our results uncover a role for MAP4K3 in the control of autophagy and reveal MAP4K3 as a central node in nutrient-sensing regulation.

## Abstract (web summary)

Amino acids stimulate cell growth and depletion in a cell activates autophagy, yet how this is regulated is unclear. Here, the authors show that MAP4K3 (also known as germinal-center kinase-like kinase) acts as an amino acid-dependent regulator of autophagy, via phosphorylation of the transcription factor EB.

## Introduction

Autophagy refers to a set of three cellular processes, i.e., macroautophagy, chaperone-mediated autophagy, and microautophagy, each of which achieve the sequestration and delivery of cytosolic cargoes to the lysosome for degradation. Macroautophagy (hereafter referred to as autophagy) is a tightly regulated cellular process by which long-lived proteins, macromolecules, and organelles are degraded^1^. Autophagy can be selective or non-selective in terms of which cargoes are directed to the lysosome for degradation and the basis for substrate selection remains an area of active research with many underlying principles yet to be elucidated. The regulation of autophagy activation and autophagosome formation, on the other hand, is better worked out, with specific protein complexes implicated in the process of initiation, nucleation, and expansion of the phagophore isolation membrane (reviewed in ref. ^2^). One critical feature of autophagy regulation is its incredibly dynamic nature, with autophagy activation status constantly responding to cellular nutrient levels and stress conditions. As autophagy-mediated protein degradation yields free amino acids for protein synthesis and energy production, amino acid depletion is a very powerful activator of autophagy. The importance of the autophagy pathway for promoting physiological processes supported by amino acids has been demonstrated in knockout (k.o.) mice lacking critical autophagy genes, as Atg5- and Atg7-null mice exhibit embryonic and neonatal lethality linked to depletion of amino acids, due to impaired protein synthesis and diminished tricarboxylic acid (TCA) cycle function^3–5^.

Mitogen-activated protein kinases (MAPKs) comprise a large family of highly conserved proteins that control a wide range of cellular processes in all eukaryotes^6^. MAP4K3, also known as germinal-center kinase-like kinase, is a member of the Ste20 sub-family of MAPKs^7^ and has been implicated in autoimmune disease via activation of protein kinase C-θ^8^, activation of c-Jun N-terminal kinase (JNK) to promote apoptosis^7^, and the amino acid-stimulated activation of the mechanistic target of rapamycin complex 1 (mTORC1), a multi-protein subunit complex consisting of the catalytic mTOR subunit, mLST8, DEPTOR, the Tti1–Tel2 complex, Raptor, and PRAS40^9^. Studies in mammalian cell lines and in *Drosophila* have shown that MAP4K3 is absolutely required for activation of mTORC1 in response to amino acids^9–11^ and amino acid levels principally determine the activation status of mTORC1^12, 13^. Furthermore, MAP4K3 is ubiquitously expressed, as MAP4K3 RNA and protein are detected in all human tissues^7, 14^. Thus, MAP4K3 probably has a central role in regulating the metabolic disposition of the cell, but nothing is known as to how MAP4K3 achieves this regulation.

We recently discovered that knock-down of MAP4K3 is sufficient to induce autophagy^15^ and so considered the current model of amino-acid-dependent autophagy regulation. According to this model, in response to amino acid stimulation, mTORC1 is recruited to the cytosolic surface of lysosomes via a physical interaction between Raptor, a set of membrane-bound lysosomal proteins known as the Ragulator complex, and the Rag GTPases, which function as heterodimers wherein the active complex consists of GTP-bound RagA or B complexed with GDP-bound RagC or D^16, 17^. When amino acids are plentiful, GATOR1, the GTPase-activating protein for Rag A/B, is inactive^18^, whereas Folliculin, the GTPase-activating protein for Rag C/D, is turned on^19^. Of the various amino acid inputs to mTORC1, leucine and arginine appear to be the most potent^20^. Leucine is sensed in the cytosol by Sestrin 1 and 2, which physically interact with and inhibit GATOR2 when leucine levels drop^21^; however, when leucine is abundant, Sestrin binding to GATOR2 is abrogated, permitting GATOR2 to promote mTORC1 activation through the Rag GTPases, possibly via its inhibition of GATOR1. Arginine is sensed in the cytosol by CASTOR1, which binds to and inhibits GATOR2 when arginine levels diminish^22^. Similar to the model for Sestrin regulation, arginine abundance promotes release of CASTOR1 from GATOR2, favoring GATOR2 activation of mTORC1^23^. The lysosomal amino acid transporter SLC38A9, a transmembrane protein, also serves as a sensor of arginine, but in the lysosomal membrane, where it binds to the Rag GTPases and Ragulator to favor mTORC1 activation upon arginine satiety^24, 25^. Bringing mTORC1 to lysosomes is critical for the activation of its kinase activity by Rheb, a lysosome-enriched GTPase that is regulated by TSC2^26^.

At the lysosome, mTORC1 directly represses autophagy by phosphorylating and inhibiting transcription factor EB (TFEB)^27–29^. TFEB is a helix-loop-helix transcription factor that recognizes a 10-base pair motif (5′-GTCACGTGAC-3′) enriched in the promoter regions of numerous lysosomal genes^30^. Activation of TFEB not only induces the expression of genes associated with lysosomal function but also transactivates genes necessary for autophagosome formation, autophagosome–lysosome fusion, and cargo degradation^31, 32^. Under conditions of amino acid satiety, TFEB interacts with active Rag GTPases, which recruit TFEB to the lysosomal surface, where mTORC1 phosphorylation of TFEB at serine 211 creates a binding site for 14-3-3, a chaperone that sequesters TFEB in the cytosol^27–29^. However, the sufficiency of mTORC1 phosphorylation of TFEB at serine 211 has been challenged by the observation that the amino-terminal region of TFEB must be present for proper regulation of TFEB subcellular localization and function^33^, although the nature of this crucial TFEB amino-terminal regulation is currently lacking.

To determine the role of MAP4K3 in the control of amino acid-mediated autophagy regulation, we generated MAP4K3 k.o. cells in this study and found that MAP4K3 k.o. is sufficient to promote TFEB nuclear localization, resulting in a remarkable upregulation of TFEB-regulated genes and productive induction of autophagy. We then examined the amino-terminal region of TFEB and noted that the phosphorylation status of the serine 3 residue of TFEB supersedes mTORC1 phosphoregulation of TFEB and autophagy. We found evidence for a direct physical interaction between MAP4K3 and TFEB, and for MAP4K3 phosphorylation of TFEB at serine 3. We also documented that the TFEB serine 3 phosphorylation is required for its inhibitory phosphorylation by mTORC1 and observed amino acid-dependent subcellular localization of MAP4K3. These results thus establish MAP4K3 as a key node in the amino acid-mediated control of autophagy and reveal MAP4K3 as a putative nutrient-sensing regulator in the cell.

## Results

### Knockout of MAP4K3 promotes autophagy induction and flux

**Fig. 1:**
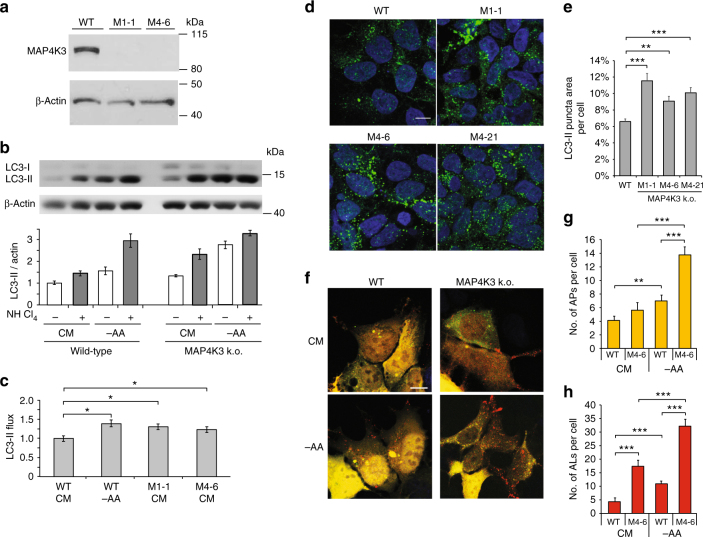
Knockout of MAP4K3 promotes autophagy induction and flux. **a** Validation of MAP4K3 knockout (k.o.) cell lines. Wild-type (WT) and HEK293A cells gene-edited with either of two different sgRNAs (M1 and M4) were lysed, and protein lysates were immunoblotted for MAP4K3. Immunoblotting of β-actin served as a loading control. **b**,** c** Knockout of MAP4K3 promotes autophagy flux. WT HEK293A cells, M1-1 MAP4K3 k.o. cells, and M4-6 MAP4K3 k.o. cells (not shown) were cultured in complete media (CM) or subjected to amino acid starvation (– AA), and remained untreated or were treated with ammonium chloride. Protein lysates were immunoblotted for LC3 and β-actin, which served as a loading control **b**. The ratio of LC3-II:actin was determined by densitometry using ImageJ and normalized to WT CM, which was arbitrarily set to 1 **c**. One-way ANOVA with post-hoc Tukey’s test; *P < 0.05. **d**,** e** Knockout of MAP4K3 promotes autophagy induction. LC-3 immunostaining of WT HEK293A cells and three different MAP4K3 k.o. cell lines, all cultured in CM **d**. Quantification of LC3 puncta area per cell area was determined using ImageJ. *n* > 100 cells per genotype. One-way ANOVA with post-hoc Tukey’s test; **P < 0.01, ***P < 0.001. **f**–**h** Knockout of MAP4K3 promotes autophagy flux. WT HEK293A cells and MAP4K3 k.o. cells were cultured in CM or amino acid starved, and were transfected with a GFP-mCherry-LC3 expression construct (**f**). Note the predominance of red puncta indicative of autolysosomes in MAP4K3 k.o. cells. Quantification of autophagosome number per cell was determined by counting yellow puncta GFP-mCherry-LC3-expressing cell (**g**). Quantification of autolysosome number per cell was determined by counting red puncta/GFP-mCherry-LC3-expressing cell (**h**). *n* > 50 cells per condition. One-way ANOVA with post-hoc Tukey’ test; **P < 0.01, ***P < 0.001. All experiments were performed in triplicate. Error bars = SEM. Scale bars = 10 μm

Knock-down of MAP4K3 is sufficient to induce autophagy^15^. However, to fully determine the role of MAP4K3 in autophagy regulation, we chose to derive MAP4K3 k.o. cell lines via CRISPR-Cas9 gene editing with guide RNAs targeting two different MAP4K3 exon sequences in HEK293 cells. This approach yielded two distinct sets of clones (M1 and M4) with frameshift mutations at either of the two targeted sites, resulting in a complete loss of MAP4K3 protein expression (Fig. 1a and Supplementary Fig. 1). To evaluate the status of the autophagy pathway in clonal MAP4K3 k.o. cell lines, we cultured MAP4K3 k.o. cells and control HEK293 cells in either nutrient replete complete media (CM) or under conditions of amino acid deprivation, and noted increased levels of LC3-II in MAP4K3 k.o. cells upon LC3 immunoblot analysis (Fig. 1b). To assess flux through the autophagy pathway, we also measured LC3-II levels after treatment with the lysosomal inhibitor ammonium chloride and documented a further increase in LC3-II levels in both MAP4K3 k.o. cells and control HEK293 cells (Fig. 1b), indicative of autophagy induction and progression to autophagosome–lysosome fusion. Calculation of LC3-II flux, based upon LC3 immunoblotting, revealed significantly increased autophagy activation in MAP4K3 k.o. cell lines in normal media in comparison with control HEK293 cells cultured under the same conditions (Fig. 1c). To corroborate these findings, we also performed LC3 immunostaining analysis of various MAP4K3 k.o. cell lines and control HEK293 cells, and observed a remarkably increased LC3 puncta formation in cells lacking MAP4K3 (Fig. 1d, e). To directly evaluate autophagy flux via immunostaining, we transfected MAP4K3 k.o. cells and control HEK293 cells with a GFP-mCherry-LC3 tandem-tagged reporter construct, quantified autophagosome and autolysosome formation in normal media and upon amino acid starvation, and observed significant increases in the numbers of autophagosomes and autolysosomes per cell in MAP4K3 k.o. cell lines (Fig. 1f-h), indicative of increased autophagy induction and flux in the absence of MAP4K3.

### MAP4K3 regulation of TFEB is upstream of mTORC1

**Fig. 2:**
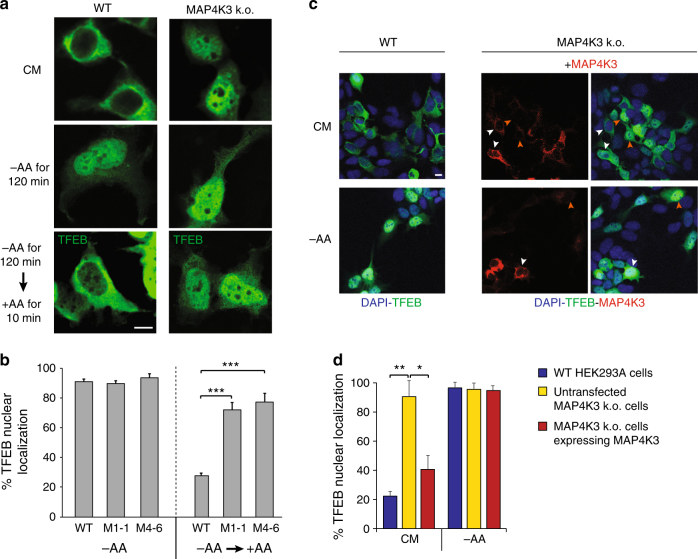
MAP4K3 regulates TFEB subcellular localization. **a** Knockout of MAP4K3 yields TFEB nuclear localization. WT HEK293A cells and MAP4K3 k.o. cells were transfected with a TFEB-FLAG expression construct and cultured in complete media (CM) or starved of amino acids for 120 min, and then restimulated with amino acids for 10 min. Here we see representative images of cells immunostained with anti-FLAG antibody. **b** Quantification of cells with predominantly TFEB nuclear localization from experiment shown in **a**. *n* > 100 cells per condition. One-way ANOVA with post-hoc Tukey’s test; ***P < 0.001. **c** TFEB nuclear localization in MAP4K3 k.o. cells is rescued by MAP4K3 expression. WT HEK293A cells and MAP4K3 k.o. cells were transfected with a TFEB-FLAG expression construct and cultured in CM or amino-acid starved, and MAP4K3 k.o. cells were co-transfected with a MAP4K3-mCherry expression construct. Cells were stained with DAPI and immunostained with anti-FLAG antibody to permit visualization of nuclei, TFEB, and MAP4K3, as indicated. TFEB remains in the cytosol in CM in WT HEK293A cells and in MAP4K3 k.o. cells transfected with the MAP4K3 vector (white arrows); however, TFEB exhibits nuclear localization in untransfected MAP4K3 k.o. cells (orange arrows). Under conditions of amino acid starvation, TFEB translocates to the nucleus in WT cells, MAP4K3-transfected MAP4K3 k.o. cells (white arrow), and untransfected MAP4K3 k.o. cells (orange arrow). **d** Quantification of cells with predominantly TFEB nuclear localization from experiment shown in **c**. *n* > 50 cells per condition. One-way ANOVA with post-hoc Tukey’s test; *P < 0.05, **P < 0.01. All experiments were performed in triplicate. Error bars = SEM. Scale bars = 10 μm

TFEB is a transcriptional activator of autophagy and is regulated by its subcellular localization^30^. As TFEB entry into the nucleus is required for transactivation of its target genes and inhibition of TFEB by mTORC1 phosphorylation restricts TFEB to the cytosol, we examined the effect of MAP4K3 loss-of-function on TFEB subcellular localization. Under conditions of amino acid deprivation, TFEB localizes to the nucleus in control HEK293 cells as well as in MAP4K3 k.o. cells (Fig. 2a, b and Supplementary Fig. 1b). When amino acids are supplied to control HEK293 cells previously subjected to amino acid starvation, TFEB no longer localizes to the nucleus but instead mostly remains in the cytosol. However, in MAP4K3 k.o. cells, the amino acid-induced sequestration of TFEB in the cytosol is dramatically blunted, as the vast majority of MAP4K3 k.o. cells display TFEB nuclear localization (Fig. 2a, b). To confirm that this difference in TFEB subcellular localization is solely attributable to MAP4K3 loss-of-function, we transfected MAP4K3 k.o. cells with a MAP4K3 expression construct, and observed rescue of amino-acid induced TFEB cytosolic sequestration in MAP4K3 k.o. cells expressing MAP4K3 (Fig. 2c, d). Failure of complete rescue upon MAP4K3 overexpression can likely be attributed to the fact that MAP4K3 induces the JNK signaling pathway and caspase activation when overexpressed, resulting in cellular stress^34^.

**Fig. 3:**
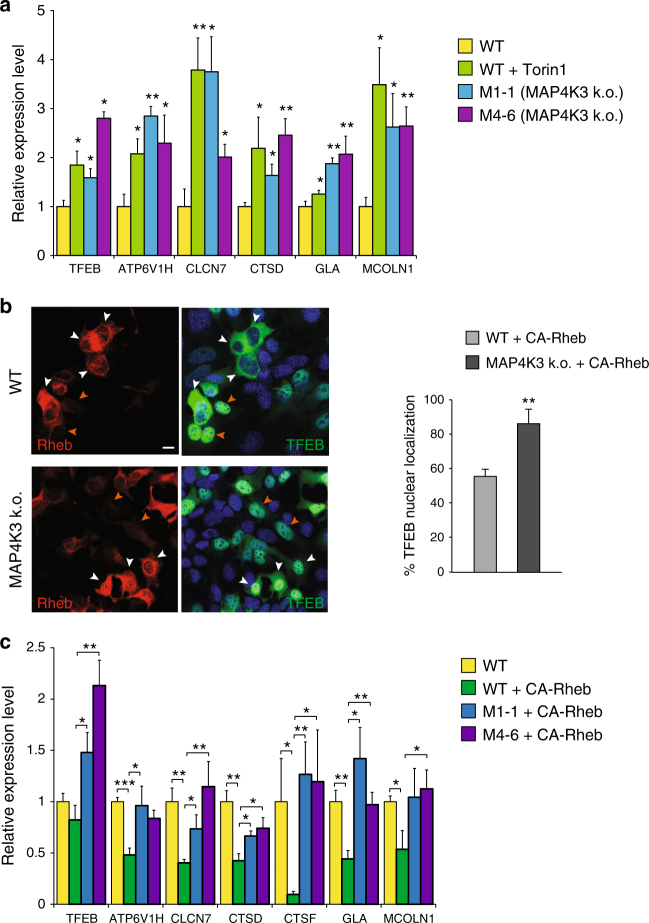
MAP4K3 regulation of TFEB is upstream of mTORC1. **a** Knockout of MAP4K3 promotes TFEB-mediated transactivation of its target genes. WT HEK293A cells, untreated, or treated with Torin1, and two different MAP4K3 k.o. cell lines were cultured in CM. Quantitative RT-PCR of isolated RNAs for these cell lines was performed for six TFEB target genes. One-way ANOVA with post-hoc Tukey’s test; *P < 0.05, **P < 0.01. **b** Activation of mTORC1 does not alter TFEB localization in MAP4K3 k.o. cells. WT HEK293A cells and MAP4K3 k.o. cells were transfected with an expression construct for constitutively active Rheb, epitope-tagged with myc, and starved of amino acids for 120 min. Although untransfected WT HEK293A cells exhibit TFEB nuclear localization (orange arrows), many WT HEK293A cells expressing constitutively active Rheb display TFEB cytosolic localization (white arrows). Although untransfected MAP4K3 k.o. cells also exhibit TFEB nuclear localization (orange arrows) as expected, most MAP4K3 k.o. cells expressing constitutively active Rheb show that TFEB still localizes to the nucleus (white arrows). Scale bar = 10 μm. Quantification of cells with predominantly TFEB nuclear localization for this experiment is shown in the adjacent graph. *n* > 50 cells per condition. **P < 0.01; two-tailed *t*-test. **c** Activation of mTORC1 does not prevent TFEB-mediated target gene activation in MAP4K3 k.o. cells. WT HEK293A cells, mock transfected, or transfected with constitutively active Rheb, and two different MAP4K3 k.o. cell lines, each transfected with constitutively active Rheb, were cultured in CM. Quantitative RT-PCR of isolated RNAs for these cell lines was performed for seven TFEB target genes. One-way ANOVA with post-hoc Tukey’s test; *P < 0.05, **P < 0.01. All experiments were performed in triplicate. Error bars = SEM

To further evaluate TFEB function in MAP4K3 k.o. cells, we measured the expression of TFEB target genes in MAP4K3 k.o. cells and in control HEK293 cells, and documented a marked induction of TFEB target genes in nutrient replete cells lacking MAP4K3—at levels comparable to those obtained upon treatment of control HEK293 cells with the mTORC1 inhibitor Torin1 (Fig. 3a). We evaluated MAP4K3 regulation of TFEB target gene expression in TFEB k.o. cells, which were derived by CRISPR/Cas9 gene editing, and confirmed that the presence of TFEB is required to yield this effect (Supplementary Fig. 2a). MAP4K3 is known to activate mTORC1 under conditions of amino acid satiety^9^, raising the question of whether MAP4K3 regulation of TFEB is upstream or downstream of mTORC1 regulation. To address this question, we repeated the amino-acid induced TFEB translocation experiment, but transfected HEK293 cells and MAP4K3 k.o. cells with the constitutively active Rheb-Q64L mutant (CA-Rheb)^35^, which potently activates mTORC1 even in the absence of amino acids (Supplementary Fig. 2b). Although many control HEK293 cells expressing CA-Rheb exhibited TFEB cytosolic localization despite amino acid starvation, most MAP4K3 k.o. cells expressing CA-Rheb retained TFEB in the nucleus upon amino acid starvation (Fig. 3b). CA-Rheb expression similarly failed to elicit a marked change in TFEB subcellular localization in MAP4K3 k.o. cells cultured in normal CM (Supplementary Fig. 3), demonstrating that MAP4K3 regulation of TFEB is a key input. To confirm the role of MAP4K3 regulation of TFEB vis-à-vis mTORC1 activation status, we also measured the expression of TFEB target genes in MAP4K3 k.o. cell lines expressing CA-Rheb and documented marked induction of TFEB target genes at levels that were significantly higher than the expression levels of TFEB target genes obtained in HEK293 cells expressing CA-Rheb (Fig. 3c). Importantly, levels of TFEB target gene expression in MAP4K3 k.o. cells were unchanged upon transfection with CA-Rheb (Supplementary Fig. 4).

### MAP4K3 regulation of TFEB interaction with mTORC1-Ragulator

**Fig. 4:**
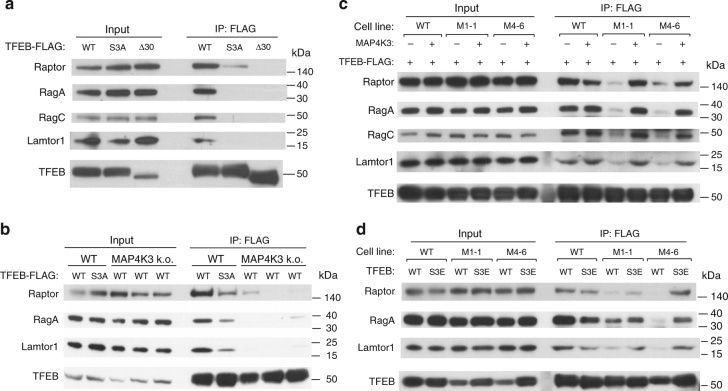
MAP4K3 and TFEB serine 3 phosphorylation are required for interaction of TFEB with the mTORC1-Rag GTPase complex. **a** TFEB serine 3 is required for its interaction with the mTORC1-Rag GTPase complex. HEK293A cells were transfected with either WT TFEB, TFEB-S3A, or TFEB-Δ30, each FLAG-tagged, and cell lysates and FLAG immunoprecipitates were subjected to immunoblotting. **b** MAP4K3 is required for TFEB interaction with the mTORC1-Rag GTPase complex. WT HEK293A cells and MAP4K3 k.o. cells were transfected with either WT TFEB or TFEB-S3A, each FLAG-tagged, and cell lysates and FLAG immunoprecipitates were subjected to immunoblotting. **c** TFEB interaction with the mTORC1-Rag GTPase complex is rescued by MAP4K3 in MAP4K3 k.o. cells. WT HEK293A cells, M1-1 MAP4K3 k.o. cells, and M4-6 MAP4K3 k.o. cells were transfected with TFEB-FLAG alone, or co-transfected with TFEB-FLAG and MAP4K3, and cell lysates and FLAG immunoprecipitates were subjected to immunoblotting. **d** TFEB phosphomimetic S3E enhances TFEB interaction with the mTORC1-Rag GTPase complex in MAP4K3 k.o. cells. WT HEK293A cells, M1-1 MAP4K3 k.o. cells, and M4-6 MAP4K3 k.o. cells were transfected with either WT TFEB or TFEB-S3E, each FLAG-tagged, and cell lysates and FLAG immunoprecipitates were subjected to immunoblotting. All experiments were performed in triplicate

Retention of TFEB in the cytosol is determined by its phosphorylation status, as phospho-TFEB complexes with 14-3-3, thereby precluding TFEB nuclear entry, whereas dephosphorylated TFEB readily translocates to the nucleus^28^. TFEB interacts with activated Rag GTPases, which promote recruitment of TFEB to the lysosomal surface, where mTORC1 phosphorylates TFEB on serine 211 to enforce its cytosolic retention and inactivation^33^. One previous study found that the first 30 amino acids of TFEB are required for TFEB localization to lysosomes and documented that mutagenesis of serine 3 and arginine 4 to alanines (S3A/R4A) completely prevented TFEB lysosomal localization^33^. To confirm the importance of the TFEB amino-terminal region for regulation of its subcellular localization and in particular the role of serine 3, we transfected control HEK293 cells with WT TFEB and confirmed robust interaction of TFEB with Raptor, Rag A, Rag C, and Lamtor1 (Fig. 4a). However, when we transfected HEK293 cells with a version of TFEB with serine 3 mutated to alanine (TFEB-S3A), or with the first 30 amino acids of TFEB deleted (TFEB-Δ30), we found that both the S3A mutation and the deletion of the first 30 amino acids of TFEB abrogated its interaction with mTORC1, the Rag GTPases, and the Ragulator complex (Fig. 4a).

The mechanistic basis for serine 3 regulation of TFEB interaction with the mTORC1 complex is yet to be determined. As serine residues are subject to phosphorylation and MAP4K3 is a kinase, we considered the possibility that MAP4K3 regulation of TFEB is occurring through phosphorylation of TFEB. To initially test this hypothesis, we evaluated the physical interaction of TFEB with mTORC1 and its associated regulatory proteins in complex with it at the lysosome. Although immunoprecipitation of TFEB provided evidence for robust interactions with Raptor, Rag A, and Lamtor1 in control HEK293 cells, interaction of TFEB with these mTORC1 complex components was dramatically reduced in two different MAP4K3 k.o. cell lines (Fig. 4b). Concern has been raised that CRISPR-Cas9 gene editing may produce off-target alterations in unrelated genes throughout the genome. Although our results were obtained in distinct MAP4K3 k.o. cell lines derived with different guide RNAs, to exclude off-target effects as a potential explanation for our findings, we repeated the TFEB interaction studies in MAP4K3 k.o. cell lines transfected with MAP4K3, and noted that exogenous MAP4K3 expression restored TFEB interaction with Raptor, Rag GTPases, and Lamtor1 in two different MAP4K3 k.o. cell lines (Fig. 4b). Noticeably, reduced interaction of TFEB-S3A with mTORC1 complex components was similarly observed in control HEK293 cells (Fig. 4c). These results indicate that MAP4K3 is required for TFEB interaction with the mTORC1 complex, and that mutation of serine 3 to an alanine, which cannot be phosphorylated, renders TFEB incapable of fully interacting with the mTORC1 complex, highlighting serine 3 as a potential site for TFEB phosphoregulation. To further evaluate this hypothesis, we expressed either wild-type TFEB or TFEB S3E, which features a phosphomimetic amino acid substitution of glutamate for serine 3, in control or MAP4K3 k.o. cells, and we found that expression of TFEB-S3E partially restored TFEB interaction with mTORC1 complex components (Fig. 4d). These findings thus establish a role for MAP4K3 and TFEB serine 3 phosphorylation in the regulation of TFEB interactions with the mTORC1 complex.

### MAP4K3 interacts with and phosphorylates TFEB at serine 3

**Fig. 5:**
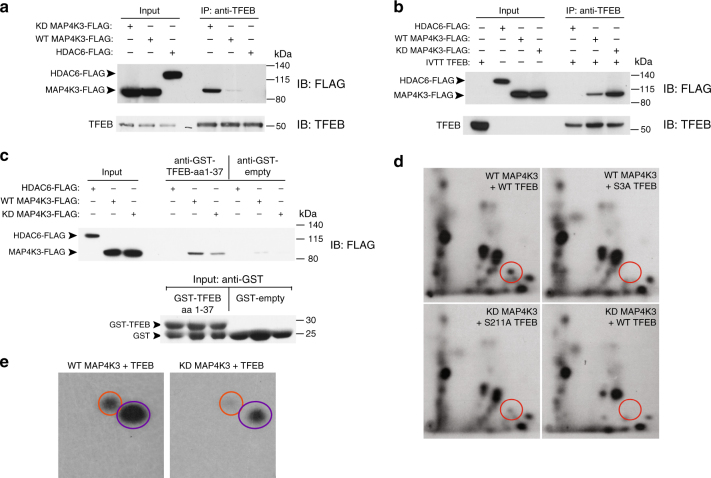
MAP4K3 phosphorylates TFEB on serine 3. **a** MAP4K3 physically interacts with TFEB. WT HEK293A cells were transfected with an expression vector for either kinase dead (KD)-MAP4K3-FLAG, WT-MAP4K3-FLAG, or HDAC6-FLAG, and cell lysates and FLAG immunoprecipitates were subjected to immunoblotting. **b** MAP4K3 directly interacts with TFEB. WT HEK293A cells were transfected with an expression vector for either HDAC6-FLAG, WT-MAP4K3-FLAG, or kinase dead (KD)-MAP4K3-FLAG, and FLAG immunoprecipitates were incubated with recombinant TFEB generated by in vitro transcription and translation (IVTT TFEB). Cell lysates and TFEB immunoprecipitates were then subjected to immunoblotting. **c** MAP4K3 directly interacts with the N-terminal 37 amino acids of TFEB. WT HEK293A cells were transfected with an expression vector for either kinase dead (KD)-MAP4K3-FLAG, WT-MAP4K3-FLAG, or HDAC6-FLAG, and FLAG immunoprecipitates were incubated with recombinant GST-TFEB amino acids 1-37 or recombinant GST alone, before mixing with GST-containing beads. Cell lysates and the eluate obtained from GST-bound fractions were subjected to anti-FLAG and anti-GST immunoblotting, as indicated. **d** MAP4K3 phosphorylates TFEB at serine 3. WT HEK293A cells were transfected with WT-MAP4K3-FLAG or KD-MAP4K3-FLAG, and either TFEB-FLAG, TFEB-S3A-FLAG, or TFEB-S211A-FLAG, as indicated. FLAG immunoprecipitates were subjected to in vitro kinase reactions with γ-P^32^-ATP, with Torin1 and the general kinase inhibitor FSBA included in the reaction mixture. Phosphopeptide mapping was performed after enzymatic digestion with thermolysin by spotting the resulting peptide mix onto cellulose thin layer chromatography plates, followed by 2D gel electrophoresis and chromatography, and finally autoradiography to visualize phospho-labeled peptides. Circles indicate location of phospho-S3-TFEB. Note the absence of phospho-S3-TFEB for TFEB-S3A and for kinase-dead (KD) MAP4K3. **e** MAP4K3 heavily phosphorylates TFEB on serines and threonines. WT HEK293A cells were transfected with WT-MAP4K3-FLAG or KD-MAP4K3-FLAG, and TFEB-FLAG, as indicated. FLAG immunoprecipitates were subjected to in vitro kinase reactions with γ-P^32^-ATP, and phospho-amino acid mapping performed by matching the resultant spots on the autoradiograph with ninhydrin-stained standards. Orange circles indicate phospho-serine, and purple circles indicate phospho-threonine. All experiments were performed in triplicate

To determine whether MAP4K3 and TFEB interact, we transfected HEK293 cells with a kinase dead version of MAP4K3 (KD-MAP4K3) and with normal MAP4K3 (WT-MAP4K3). When we immunoprecipitated endogenous TFEB and immunoblotted for MAP4K3, we detected a physical interaction between TFEB and MAP4K3, but not between TFEB and an unrelated control protein, HDAC6, with the identical epitope tag (Fig. 5a). Interestingly, the interaction between TFEB and KD-MAP4K3 was significantly stronger than the interaction between TFEB and WT-MAP4K3, suggesting that MAP4K3 kinase activity dictates the nature of its interaction with TFEB, such that failure of KD-MAP4K3 to phosphorylate substrate may favor an extended physical interaction. To determine whether the MAP4K3–TFEB interaction is direct, we generated in vitro transcribed and translated TFEB protein, and performed pull-down assays with purified FLAG-tagged WT-MAP4K3, KD-MAP4K3, or HDAC6. WT-MAP4K3 and KD-MAP4K3 were both pulled down with an anti-TFEB antibody, but the pull-down of KD-MAP4K3 was clearly stronger (Fig. 5b). Finally, to determine whether the physical interaction of TFEB with MAP4K3 involves the amino-terminal region of TFEB, we generated a recombinant amino-terminal fragment of TFEB (TFEB:aa1-37) linked to glutathione *S*-transferase (GST) and documented that TFEB:aa1–37 is capable of pulling down WT-MAP4K3 or KD-MAP4K3 (Fig. 5c). These results provide evidence for a direct interaction between MAP4K3 and TFEB, and indicate that the first 37 amino acids of TFEB are critical for this physical interaction.

As MAP4K3 is a kinase and our results implicate the serine 3 residue of TFEB as a key site for its phosphoregulation, we sought to test the hypothesis that MAP4K3 is phosphorylating TFEB at serine 3. Although mass spectrometry is a powerful method for mapping phosphorylation sites, we did not employ this approach, as trypsin digestion of TFEB is predicted to yield a tiny four-amino-acid amino-terminal fragment, which would not be detectable. Rather, to determine whether MAP4K3 can phosphorylate TFEB, we performed in vitro phosphopeptide mapping in reaction mixtures containing the mTOR inhibitor Torin1 and a general kinase inhibitor. After two-dimensional gel fractionation, we observed a phosphopeptide fragment that was present upon co-incubation of WT-MAP4K3 with either WT TFEB or TFEB-S211A, but absent upon co-incubation of WT-MAP4K3 with TFEB-S3A, or KD-MAP4K3 with WT TFEB under these phosphorylation conditions (Fig. 5d and Supplementary Fig. 5). Phospho-amino acid analysis of these peptides confirmed that the level of phosphoserine was lower for TFEB in the KD-MAP4K3 + TFEB reaction, in comparison with the WT-MAP4K3 + WT-TFEB reaction (Fig. 5e). These findings thus illustrate that MAP4K3 may phosphorylate TFEB on serine 3.

### Serine 3 phosphorylation precedes TFEB serine 211 phosphorylation

**Fig. 6:**
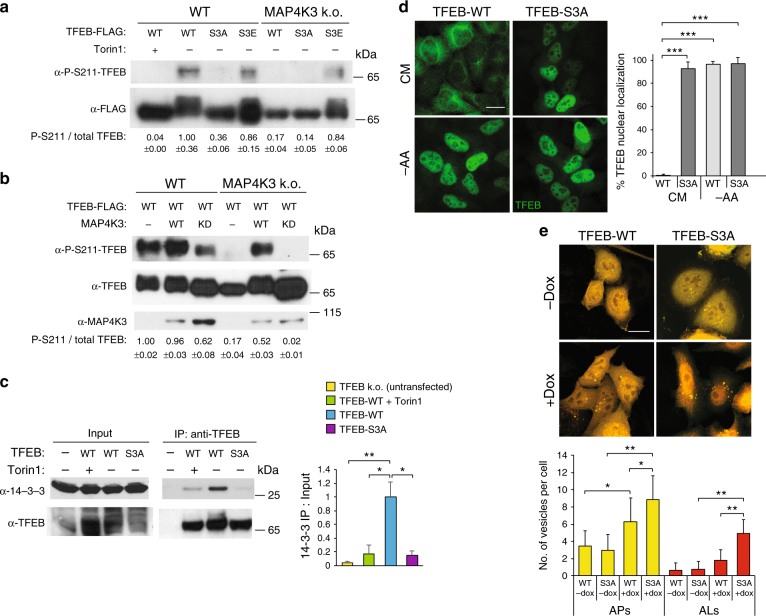
Phosphorylation of TFEB at serine 3 is a key determinant of TFEB cellular regulation and autophagy function. **a** TFEB serine 3 phosphorylation is required for mTORC1 phosphorylation at serine 211. WT HEK293A and MAP4K3 k.o. cells were transfected with TFEB-FLAG, TFEB-S3A-FLAG, or TFEB-S3E-FLAG, and Torin1 treated. FLAG immunoprecipitates were immunoblotted and TFEB serine 211 phosphorylation as a fraction of total TFEB was quantified by densitometry. **b** MAP4K3 phosphorylation of TFEB is required for mTORC1 phosphorylation at serine 211. WT HEK293A and MAP4K3 k.o. cells were transfected with TFEB-FLAG and either no MAP4K3 (--), WT-MAP4K3, or KD-MAP4K3. FLAG immunoprecipitates were immunoblotted, and TFEB serine 211 phosphorylation as a fraction of total TFEB was quantified by densitometry. **c** TFEB serine 3 phosphorylation is required for interaction with 14-3-3. TFEB k.o. cells were transfected with no TFEB (--), inducible TFEB-WT-FLAG, or inducible TFEB-S3A-FLAG, and Torin1 treated, whereas all cells received doxycycline to induce TFEB-WT or TFEB-S3A expression. Cell lysates and TFEB immunoprecipitates were immunoblotted and immunoprecipitated 14-3-3 was quantified by densitometry. One-way ANOVA with post-hoc Tukey’s test; *P < 0.05, **P < 0.01. **d** TFEB serine 3 phosphorylation regulates TFEB nuclear localization. TFEB k.o. cells were transfected with inducible TFEB-WT-FLAG or TFEB-S3A-FLAG, and cultured in CM or amino-acid starved (– AA). Under amino acid deprivation, TFEB localizes to nucleus, regardless of serine 3 status; however, upon amino acid satiety, mutation of TFEB serine 3 to phospho-resistant alanine prevents retention of TFEB in the cytosol. Quantification of TFEB nuclear localization to right. *n* > 100 cells per condition. One-way ANOVA with post-hoc Tukey’s test; ***P < 0.001. **e** TFEB serine 3 phosphorylation regulates autophagy activation. TFEB k.o. cells were transfected with GFP-mCherry-LC3 and either inducible TFEB-WT-FLAG or TFEB-S3A-FLAG, and cultured in CM and doxycycline, as indicated. Note the red puncta indicative of autolysosomes in cells expressing TFEB-S3A. Autophagosome number per cell was determined by counting yellow puncta per GFP-mCherry-LC3-expressing cell, and autolysosome number per cell was determined by counting red puncta per GFP-mCherry-LC3-expressing cell. *n* > 50 cells per condition. One-way ANOVA with post-hoc Tukey’s test; *P 0*< *0.05, **P < 0.01. All experiments performed in triplicate. Error bars = SEM. Scale bars = 20 μm

mTORC1 phosphorylation of TFEB at serine 211 is viewed as a crucial regulatory event for TFEB repression^27, 28^. To determine the regulatory relationship between MAP4K3 phosphorylation of serine 3 and mTORC1 phosphorylation of serine 211, we examined the effect of TFEB serine 3 phosphorylation status upon TFEB serine 211 phosphorylation. Immunoblotting analysis of control HEK293 cells transfected with either normal TFEB or TFEB-S3A revealed a remarkable reduction in TFEB-S3A serine 211 phosphorylation that was comparable to the reduction in TFEB serine 211 phosphorylation with Torin1 treatment (Fig. 6a). When we performed TFEB phosphoserine 211 immunoblotting on MAP4K3 k.o. cells transfected with either normal TFEB, TFEB-S3A, or TFEB S3E, we only detected TFEB serine 211 phosphorylation in MAP4K3 k.o. cells transfected with the phosphomimetic TFEB S3E mutant (Fig. 6a), suggesting that serine 3 phosphorylation is required for subsequent phosphorylation of TFEB serine 211 by mTORC1. To confirm that MAP4K3 phosphorylation of serine 3 dictates the ability of mTORC1 to phosphorylate TFEB at serine 211, we performed an additional TFEB phosphoserine 211 immunoblotting experiment in control HEK293 cells and MAP4K3 k.o. cells transfected with normal TFEB in combination with either WT-MAP4K3 or KD-MAP4K3. We found that the expression of WT-MAP4K3 in MAP4K3 k.o. cells rescued TFEB serine 211 phosphorylation, but the expression of KD-MAP4K3 in MAP4K3 k.o. cells did not yield appreciable TFEB serine 211 phosphorylation (Fig. 6b). We also noted that KD-MAP4K3 expression in control HEK293 cells that possess endogenous MAP4K3 resulted in a decrease in TFEB serine 211 phosphorylation, probably reflecting a dominant-negative effect, as MAP4K3 requires transautophosphorylation at serine 170 within its kinase enzymatic domain for full activation^36^. Taken together, these results indicate that MAP4K3 kinase activity occurs before and is necessary for TFEB serine 211 phosphorylation by mTORC1.

### TFEB serine 3 phosphorylation regulates autophagy activation

To assess the physiological relevance of TFEB serine 3 phosphorylation for regulation of autophagy, we generated tetracycline-inducible TFEB-WT and TFEB-S3A expression constructs. We then created TFEB k.o. cell lines stably transfected with either inducible TFEB-WT or inducible TFEB-S3A, and confirmed inducible expression of TFEB at close to endogenous levels to validate the utility of these cell lines (Supplementary Fig. 6a). To establish the regulatory significance of the serine 3 phosphorylation for TFEB repression, we examined the role of TFEB serine 3 phosphorylation in dictating the interaction of TFEB with 14-3-3, as mTORC1 phosphorylation of TFEB at serine 211 has been shown to promote TFEB binding to 14-3-3 and its sequestration in the cytosol^27, 28^. We found that induction of TFEB expression in TFEB k.o. HeLa cells cultured in normal media resulted in a productive interaction between TFEB and 14-3-3, based upon 14-3-3 immunoblot analysis of TFEB immunoprecipitates (Fig. 6c). However, 14-3-3 immmunoblot analysis of TFEB immunoprecipitates prepared from TFEB k.o. HeLa cells expressing TFEB-S3A protein yielded barely detectable signal, akin to results obtained in TFEB k.o. cells induced to express normal TFEB but in the presence of the mTORC1 inhibitor Torin1 (Fig. 6c). To further assess the role of serine 3 phosphorylation in dictating TFEB subcellular localization and function, we cultured TFEB k.o. cells induced to express either normal TFEB or TFEB-S3A in CM or in media lacking amino acids, and then performed TFEB immunostaining analysis to examine subcellular localization. We observed nearly complete nuclear localization of TFEB when cells were starved of amino acids, as expected, but documented nearly complete nuclear localization of TFEB-S3A in amino acid-replete media, in striking contrast to complete cytosolic localization of TFEB-WT in amino acid-replete media (Fig. 6d). These results confirm that the phosphorylation status of serine 3 of TFEB is the primary determinant of its subcellular localization.

As overexpression of TFEB is sufficient to induce productive autophagy^32^, we next tested the effect of TFEB serine 3 phosphorylation on autophagy activation by expressing GFP-mCherry-LC3 in TFEB k.o. cells stably transfected with inducible TFEB-WT or inducible TFEB-S3A. When we treated these TFEB k.o. cells with doxycycline and monitored autophagic flux, we detected a much more robust activation of autophagy in TFEB k.o. cells expressing TFEB-S3A in comparison with TFEB k.o. cells expressing normal TFEB, based upon our observation of significantly greater numbers of autophagosomes and autolyosomes in the HeLa cells expressing TFEB-S3A (Fig. 6e). These results demonstrate that TFEB serine 3 phosphorylation determines TFEB function and autophagy activation status. To further corroborate the physiological relevance of increased autophagy engagement in TFEB-S3A expressing TFEB k.o. cells, we compared cell growth between induced and uninduced TFEB k.o. cells expressing either normal TFEB or TFEB-S3A, and noted that TFEB k.o. cells induced to express TFEB-S3A exhibited significantly reduced cellular proliferation over time (Supplementary Fig. 6b), culminating in markedly reduced cell numbers at 72 h after doxycycline induction (Supplementary Fig. 6c). Impaired cell proliferation of TFEB-S3A-expressing cells is consistent with the greater catabolic disposition of these cells, which display elevated autophagy pathway activation.

### MAP4K3 localizes to lysosomes upon amino acid starvation

**Fig. 7:**
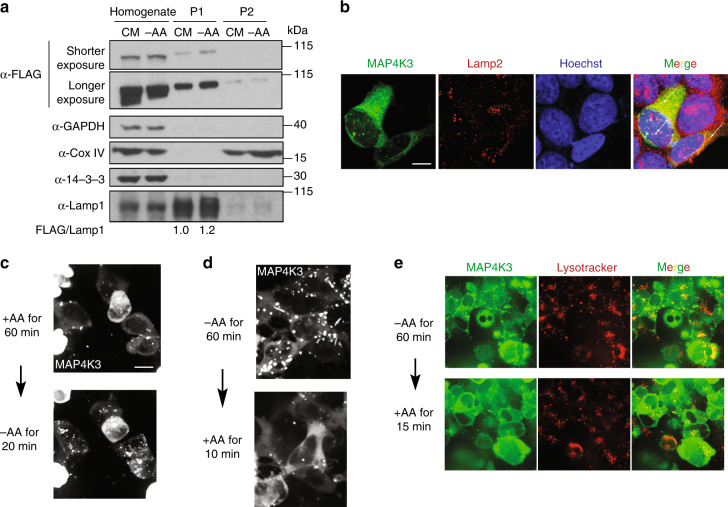
MAP4K3 exhibits lysosomal localization. **a** HEK293A cells were transfected with FLAG-tagged MAP4K3 for 16 h, then cultured in complete media (CM) or subjected to amino acid starvation (– AA) for 1 h. Cells were subjected to subcellular organelle fractionation via sucrose gradient density ultracentrifugation. The whole homogenate and isolated lysosomal fractions (P1 or P2) were collected for immunoblotting analysis, as indicated. Densitometry of MAP4K3 and Lamp1 was performed on the immunoblot of the P1 lysosomal fraction to quantify MAP4K3 in the lysosomal fraction, normalized to Lamp1. The relative ratio of MAP4K3 in the P1 fraction for CM-cultured HEK293A cells and amino-acid starved HEK293A cells is given below their respective lanes, with MAP4K3 in CM-cultured HEK293A cells arbitrarily set to 1. **b** HEK293A cells were transfected with MAP4K3-mNeonGreen and maintained under conditions of amino acid satiety for at least 60 min, before being switched to media lacking amino acids for 60 min, after which cells were fixed and immunostained for Lamp2. Note numerous puncta (arrows indicate representative examples) revealing colocalization of MAP4K3 with Lamp2. Scale bar = 10 μm. **c** HEK293A cells were transfected with MAP4K3-mNeonGreen and maintained under conditions of amino acid satiety for at least 60 min, before being switched to media lacking amino acids. Note the marked increase in MAP4K3 localization to cytosolic puncta upon amino acid starvation. Scale bar = 10 μm (see Supplementary Movie 1 for live cell imaging). **d** HEK293A cells were transfected with MAP4K3-mNeonGreen and starved of amino acids for 60 min, before being switched to amino acid-replete media. Note the MAP4K3 disassociation from cytosolic puncta to diffuse cytosolic localization upon supplying amino acids. Scale bar = 10 μm. **e** HEK293A cells were transfected with MAP4K3-mNeonGreen, treated with Lysotracker Red, and starved of amino acids for 60 min, before being switched to amino acid-replete media. Note the prominent MAP4K3 colocalization with Lysotracker Red in cytosolic puncta during amino acid starvation, then upon amino acid supplementation, and MAP4K3 movement from cytosolic puncta and Lysotracker Red colocalization to a more diffuse cytosolic localization as well. Scale bar = 10 μm (see Supplementary Movie 2 for live-cell imaging). All experiments were performed in triplicate

As our findings indicate that MAP4K3 is a key node in autophagy regulation, an important question is: how does amino acid starvation prevent MAP4K3 repression of TFEB in the cytosol? To address this question, we examined the subcellular localization of MAP4K3 by performing a subcellular fractionation of HEK293A cells expressing FLAG-tagged MAP4K3, employing a protocol that permitted isolation of a gradient fraction highly enriched for lysosomes (Supplementary Fig. 7). Immunoblotting confirmed enrichment for lysosomes in the P1 fraction, based upon the presence of abundant Lamp1 and the absence of cytosolic and mitochondrial proteins (Fig. 7a). Immunoblotting for MAP4K3 revealed that MAP4K3 is abundant in the lysosome-enriched P1 fraction, and that MAP4K3 abundance in the lysosomal fraction is increased when HEK293A cells are subjected to amino acid starvation (Fig. 7a). To further examine the subcellular localization of MAP4K3, we transfected nutrient-replete HEK293A cells with MAP4K3-mNeonGreen and then switched the HEK293A cells expressing MAP4K3-mNeonGreen to media lacking amino acids, which resulted in pronounced colocalization of MAP4K3 with Lamp2 in discrete cytosolic puncta (Fig. 7b). We also performed live-cell imaging of MAP4K3 subcellular localization in HEK293A cells subjected to amino acid starvation and found that upon amino acid starvation, MAP4K3 transitions from a diffuse cytosolic appearance to discrete localization into cytosolic puncta (Fig. 7c and Supplementary Movie 1). Furthermore, upon resupply of amino acids to amino acid-starved HEK293A cells, MAP4K3 no longer remains in cytosolic puncta but instead returns to a diffuse cytosolic localization (Fig. 7d). Formation of cytosolic puncta containing MAP4K3 upon amino acid depletion was not restricted to HEK293A cells, but was also documented in primary retinal pigmented epithelial (RPE) cells and HEK293T cells (Supplementary Fig. 8). To verify the nature of the cytosolic puncta to which MAP4K3 localizes upon amino acid starvation, we performed live-cell imaging and observed colocalization of MAP4K3 cytosolic puncta with Lysotracker Red (Fig. 7e and Supplementary Movie 2). When we supplied amino acids to starved HEK293A cells, we noted that MAP4K3 lysosomal localization decreased within minutes (Fig. 7e and Supplementary Movie 2).

**Fig. 8:**
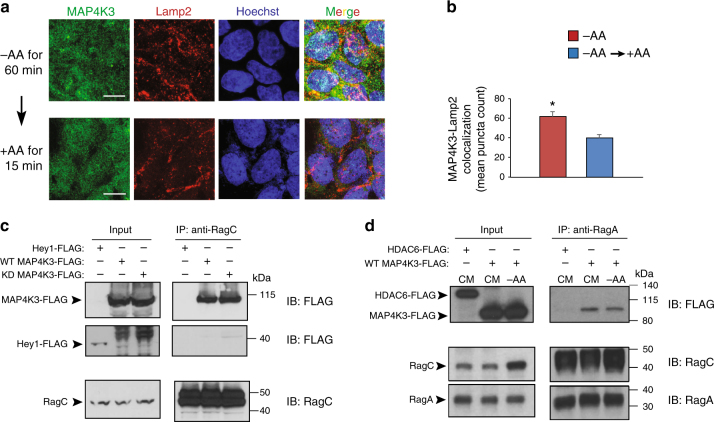
MAP4K3 preferentially localizes to lysosomes upon amino acid depletion and interacts with Rag GTPases. **a** HEK293A cells were starved of amino acids for 60 min and then switched to amino acid-replete media for 15 min, after which cells were fixed and immunostained for endogenous MAP4K3 and Lamp2. It is noteworthy that extensive MAP4K3–Lamp2 colocalization upon amino acid starvation diminishes with resupply of amino acids. Scale bar = 10 μm. **b** Quantification of MAP4K3–Lamp2 colocalization in **a**. We counted the number of colocalized puncta in 10 cells per field for 3 fields per condition, performed in triplicate, and determined the mean puncta count per condition. *P < 0.05; two-tailed *t*-test. **c** MAP4K3 physically interacts with RagC. WT HEK293A cells were transfected with an expression vector for Hey1-FLAG, WT-MAP4K3-FLAG, or kinase dead (KD)-MAP4K3-FLAG as indicated, and cell lysates and RagC immunoprecipitates were subjected to immunoblotting. **d** MAP4K3 interaction with RagA is dependent on amino acid status. WT HEK293A cells cultured in complete media (CM) or subjected to amino acid starvation (– AA) were transfected with an expression vector for either HDAC6-FLAG or WT-MAP4K3-FLAG as indicated, and cell lysates and RagA immunoprecipitates were subjected to immunoblotting. All experiments were performed in triplicate

To confirm the physiological relevance of MAP4K3 localization to lysosomes and the effect of nutrient status upon MAP4K3 subcellular localization, we again subjected HEK293A cells to amino acid starvation, but this time immunostained for endogenous MAP4K3 and Lamp2, noting extensive colocalization of MAP4K3 with Lamp2 in punctate structures (Fig. 8a). When we resupplied amino acids, we observed a significant reduction in this MAP4K3–Lamp2 colocalization (Fig. 8a-b). The recruitment of TFEB and mTORC1 to the lysosome involves the Rag GTPases, whose activation is dictated by the amino acid status of the cell^23^. To determine whether MAP4K3 association with the lysosome might involve the Rag GTPases, we performed a series of immunoblots and documented a physical interaction between MAP4K3 and RagC (Fig. 8c). To evaluate the effect of amino acid status on the interaction of MAP4K3 with the Rag GTPases, we performed co-immunoprecipitation of MAP4K3 with RagA in HEK293A cells grown under nutrient replete conditions or under conditions of amino acid deprivation. We found that the interaction of MAP4K3 with RagA was comparable in amino acid-starved HEK293A cells and in nutrient-replete HEK293A cells (Fig. 8d), indicating that other components of the amino acid sensing circuitry likely regulate MAP4K3 recruitment to lysosomes.

## Discussion

Given the tight linkage between amino acid supply and the capacity of the cell to survive and support anabolic growth, amino acid sensing has emerged as a key determinant of autophagy status. Over the last decade, our understanding of how amino acid satiety regulates autophagy has advanced dramatically and has led to a model wherein certain amino acids are sensed in the cytosol and lysosome (reviewed in ref. ^37^). These sensors, when amino acid stimulated, promote the activation of the Rag GTPases that recruit the mTORC1 complex to the lysosome, and there interact with and are regulated by a set of membrane-bound proteins known as the Ragulator complex. The mTORC1-Rag GTPase-Ragulator complex, once at the lysosome, places mTORC1 in close proximity to Rheb, which thereby activates it. To fully achieve effective autophagy repression when amino acids are abundant, the Rag GTPases recruit TFEB to the lysosomal surface, where activated mTORC1 resides, to promote mTORC1 inhibition of TFEB^32, 33^. According to this model, once mTORC1 phosphorylation of TFEB at serine 211 occurs, TFEB leaves the lysosomal surface and is bound by 14-3-3 in the cytosol, which renders TFEB sequestered and inactive—until the metabolic disposition or stress level of the cell changes.

**Fig. 9:**
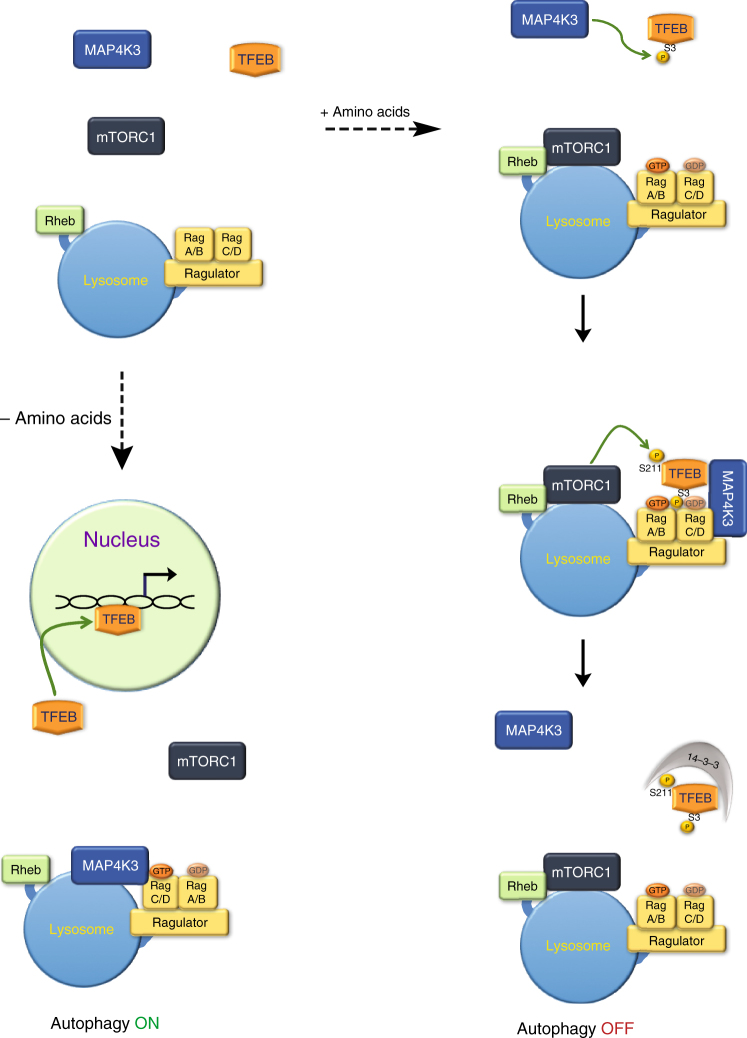
Model for MAP4K3 regulation of TFEB activation-dependent autophagy. Right: when amino acids are abundant, MAP4K3 phosphorylates TFEB on serine 3 in the cytosol. TFEB serine 3 phosphorylation enables the Rag GTPases to recruit TFEB, which may still be in complex with MAP4K3, to the surface of the lysosome via the interaction of Rag GTPases with the Ragulator complex. Recruitment of TFEB to the lysosomal surface facilitates mTORC1 interaction with TFEB and mTORC1 phosphorylation of TFEB on serine 211. Upon serine 211 phosphorylation, TFEB is released from the lysosome to the cytosol, where 14-3-3 binds to TFEB and retains inactive TFEB sequestered in the cytosol. Left: when amino acids are scarce, MAP4K3 localizes to the lysosome and TFEB is thus no longer phosphorylated, permitting TFEB to translocate into the nucleus and activate the expression of genes that promote autophagy-lysosome pathway function

We recently determined that MAP4K3 knock-down is sufficient to induce productive autophagy^15^. As MAP4K3 is an upstream regulator in the amino acid response pathway^9^, we considered the existing model for amino acid-dependent autophagy regulation and the basis for TFEB repression, as the amino acid-induced translocation of TFEB to the lysosome depends upon the amino-terminal region of TFEB^33^, which suggests that TFEB inhibition is not entirely mTORC1-dependent. We derived two different MAP4K3 k.o. cell lines and found that MAP4K3 absence yielded TFEB nuclear localization despite amino acid abundance. We generated phosphoresistant and phosphomimetic mutations of the serine 3 residue in TFEB, and documented that MAP4K3 presence and TFEB serine 3 phosphorylation are required for the interaction of TFEB with the mTORC1-Rag GTPase-Ragulator complex at the lysosome. We discovered that when amino acids are plentiful, MAP4K3 and TFEB physically interact, and MAP4K3 may phosphorylate TFEB at serine 3; this TFEB serine 3 phosphorylation appears necessary for mTORC1’s inhibitory phosphorylation at serine 211 of TFEB. We also determined that amino acid levels affect MAP4K3 subcellular localization, as amino acid depletion favors MAP4K3 lysosomal localization. Our findings support a model of amino acid-mediated autophagy regulation where MAP4K3 is acting upstream of mTORC1 in the control of TFEB localization and activation, and MAP4K3 subcellular localization itself is influenced by amino acid levels (Fig. 9). However, although our results indicate that MAP4K3 initiates TFEB repression, MAP4K3 also promotes robust mTORC1 activation upon amino acid stimulation^9–11^; hence, MAP4K3 and mTORC1 must ultimately work together to achieve robust suppression of autophagy (Fig. 9). Indeed, when we compare TFEB target gene induction achieved in MAP4K3 k.o. cells expressing CA-Rheb with TFEB target gene induction in MAP4K3 k.o. cells alone, we note that TFEB target gene expression is lower in the former situation (Fig. 3c), suggesting that mTORC1 inhibition contributes to the robust autophagy activation observed in MAP4K3 k.o. cells alone (Fig. 3a). Undoubtedly, the regulatory interactions between MAP4K3, mTORC1, and TFEB are likely to be complex, as a recent study found that TFEB paradoxically promotes mTORC1 activation through the induction of RagD, which facilitates mTORC1 localization to the lysosome^38^. Although we documented a physical interaction between MAP4K3 and the Rag GTPases, interaction of MAP4K3 with the Rag GTPases did not appear to be amino-acid dependent; hence, the regulation of MAP4K3 recruitment to lysosomes likely involves other components of the amino acid-sensing machinery. Our recognition of MAP4K3 as a key node in the regulation of autophagy in response to amino acids, however, underscores its role as a central player in nutrient sensing in the cell.

In 2007, MAP4K3 was identified as a regulator of mTORC1 activation in response to amino acid satiety in an RNA interference screen in *Drosophila*, and was shown to be required for amino acid activation of mTORC1 in mammalian cell lines^9^. Initial studies focused on the MAP4K3 fly ortholog *happyhour* (*hppy*) and revealed that *hppy* flies exhibit defects in wing cell growth, suggestive of impaired mTORC1 activation^10^. Although MAP4K3 loss-of-function phenotypes in *Drosophila* were attributed to impaired mTORC1 activation, our results implicate overactive autophagy in the retarded growth, reduced size, and markedly decreased fat mass observed in flies with low levels of MAP4K3 expression, although reduced cell growth and size in the context of MAP4K3 loss-of-function likely also results from decreased anabolic function due to mTORC1 inhibition. Our observation of increased autophagy flux in combination with reduced cell growth in cell lines expressing only the TFEB S3A isoform are consistent with a role for over-exuberant autophagy activation in the reduced cell growth phenotype. Additional evidence for the physiological importance of MAP4K3 regulation of autophagy comes from studies of certain cancers, where decreased or absent expression of MAP4K3 has been documented^39, 40^, and an extensive literature has indicated a role for increased autophagy activation in supporting the altered metabolism of thriving cancer cells^41^. MAP4K3 repression of autophagy may also contribute to the development of autoimmune diseases, such as rheumatoid arthritis or systemic lupus erythematosus, as autoimmune disease patients exhibit elevated expression of MAP4K3^42^, and k.o. of MAP4K3 in mice protects against experimental autoimmune encephalomyelitis^8^, a series of findings that are reminiscent of the association between a Atg16L variant yielding impaired autophagy function and increased risk for Crohn’s disease in humans^43^.

Our discovery of MAP4K3 as a central regulator of autophagy places it upstream of mTORC1 regulation at the lysosome. How MAP4K3 is activated by amino acids remains to be determined, as MAP4K3 has not yet been linked to any of the identified amino acid sensors or signal transducers. Defining the basis for MAP4K3 sensing of amino acid satiety and determining how MAP4K3 is trafficked will be the focus of further study. MAP4K3 is present in the cytosol and mediates the regulation of TFEB there through a direct physical interaction with TFEB, and it is the serine 3 phosphorylation that is required for TFEB’s subsequent interaction with the Rag GTPases that recruit it to the lysosome. Hence, MAP4K3-controlled recruitment of TFEB to lysosomes likely precedes the negative regulation of TFEB by the mTORC1 complex. The regulation of TFEB reactivation after MAP4K3 and mTORC1 phosphoinhibition is not entirely clear, but may involve calcium signaling from lysosomes, as release of calcium from lysosomes can activate the phosphatase calcineurin, which dephosphorylates TFEB to promote its nuclear localization^44^.

As mTORC1 activity is determined by a vast array of inputs, and autophagy activation status is dictated by various anabolic and catabolic inputs, MAP4K3 may represent an appealing target for pharmacological modulation. Indeed, although inhibition of mTORC1 can upregulate TFEB, mTORC1 is not an ideal drug target, due to its central role in regulating cell growth and macromolecule synthesis, as long-term mTORC1 inhibition results in immunosuppression and impaired wound-healing^45^. MAP4K3 inhibition could thus prove to be a highly effective therapy for diseases where enhanced autophagy activation would be beneficial, including neurodegenerative disorders, lysosomal storage diseases, and possibly autoimmune disorders. Hence, identification of MAP4K3 as an upstream regulator of autophagy offers an attractive target for therapeutically enhancing the clearance of protein aggregates and dysfunctional organelles.

## Methods

### Materials and reagents

HEK293A cells (Thermo Fisher R70507) and HeLa cells (ATCC CCL-2) were grown in Dulbecco’s modified Eagle’s medium (DMEM) media with 10% fetal bovine serum (FBS). For amino acid deprivation (– AA), cells were treated with Earle’s balanced salt solution for 2 h. Restimulation was performed in by adding DMEM with 10% FBS for 10 min. Transfections were performed using Lipofectamine 2000, according to the manufacturer’s instructions (Invitrogen). For quantitative reverse transcriptase-PCR experiments, transfection was performed with 2.4 μg of DNA per 10 cm^2^ of cells. For immunofluorescence experiments, transfection was performed with 0.08 μg of DNA per 0.7 cm^2^ of cells. After 6 h, the media was replaced. When indicated, cells were treated with 250 nM Torin1 for 2 h.

### Generation of MAP4K3 and TFEB k.o. cells

The 20-nucleotide guide sequences targeting human TFEB and MAP4K3 were designed using the CRISPR design tool at http://crispr.mit.edu/^46^ and cloned into a bicistronic expression vector (pX330) containing human codon-optimized Cas9 and RNA components (Addgene, 42230).

The guide sequences targeting Exon 1 of human MAP4K3 and Exon 3 of TFEB are as follows:

MAP4K3: 5′–TACCTTGTAGACGTCGCCGT–3′

TFEB: 5′–GAGTACCTGTCCGAGACCTA–3′

The single guide RNAs in the pX330 vector (1 µg) were mixed with enhanced green fluorescent protein (EGFP) (0.1 µg; Clontech) and co-transfected into HEK293A (for MAP4K3) or HeLa (for TFEB) cells using Lipofectamine 2000 (Life Technologies) according to the manufacturer’s instructions. Twenty-four hours post transfection, the cells were trypsinized, washed with phosphate-buffered saline (PBS), and re-suspended in fluorescence-activated cell sorting (FACs) buffer (PBS, 5 mM EDTA, 2% FBS, and Pen/Strep). GFP-positive cells were single-cell sorted by FACs (UCSD; Human Embryonic Stem Cell Core, BDInflux) into 96-well plate format into DMEM containing 20% FBS and 50 µg ml/L penicillin/streptomycin. Single clones were expanded, and screened for MAP4K3 and TFEB by protein immunoblotting. Genomic DNA was purified from clones using the DNeasy Blood & Tissue Kit (QIAGEN, 69504), and the region surrounding the protospacer adjacent motif was amplified with Phusion High-Fidelity DNA Polymerase (New England Biolabs, M0530) using the following primers:

MAP4K3:

Forward: 5′–GGAGCCGGGTGATTGTGA–3′

Reverse: 5′–AGAAGGGAGGTGGCAAAAAT–3′

TFEB:

Forward: 5′–CGTCACGCATAGGGTTGC–3′

Reverse: 5′–CGTCCAGACGCATAATGTTG–3′

PCR products were purified using the QIAquick PCR Purification Kit (QIAGEN, 28104) and cloned using the TOPO TA Cloning (ThermoFisher, K457502). To determine the specific mutations for the individual alleles, at least 10 bacterial colonies were expanded and the plasmid DNA purified and sequenced.

### Creation of doxycycline inducible WT and S3A-TFEB HeLa cells

Generation of doxycycline-inducible cell lines was accomplished by generating multi-cistronic pCAM vectors utilizing the TetOn3G (Clontech) expression cassette. Vectors are expressing puroR-2A-TetOn3G under the control of the chicken β-actin promoter or EGFP-2a-TFEB(WT)-FLAG or TFEB(S3A)-FLAG under the control of the Tetracycline Responsive Element promoter. The plasmids were linearized and transfected into the TFEB k.o. HeLa cells using Lipofectamine 2000 to generate stable cell lines. Cells were selected using puromycin and FACs sorted (UCSD; Human Embryonic Stem Cell Core, BD Influx) for GFP-expressing cells post-doxycycline addition with very high expressing cells excluded from the sort, to ensure near-endogenous expressing cell lines. Dilutions of doxycycline treatment followed by immunoblotting for TFEB was performed to determine the doxycycline concentration that would induce the WT or S3A TFEB expression in cells lines at near-endogenous levels. In all experiments, WT TFEB cells were treated with 20 ng/μL doxycycline and S3A TFEB cell lines were treated with 200 ng/μL doxycline.

### Cell lysis and immunoprecipitation

Cells were rinsed twice with ice-cold PBS and lysed in ice-cold lysis buffer (25 mM HEPES-KOH pH 7.4, 150 mM NaCl, 5 mM EDTA, 1% Triton X-100 40 mM, 1 tablet of EDTA-free protease inhibitors (Roche, 11873580001) per 10 mL of lysis buffer, and 1 tablet of PhosStop phosphatase inhibitor (Roche, 4906845001), as necessary. The soluble fractions from cell lysates were isolated by centrifugation at 8,000 r.p.m. for 10 min in a microfuge. Protein lysates were quantified using Pierce BCA Protein Assay Kit (ThermoFisher, 23225) following the manufacturer’s protocol. For immunoprecipitations, primary antibodies were incubated with Dynabeads (Invitrogen) overnight, then washed with sterile PBS. Antibodies bound to Dynabeads were then incubated with lysates with rotation for 2 h at 4 °C. Immunoprecipitates were washed three times with lysis buffer. Immunoprecipitated proteins were denatured by the addition of 20 µL of sample buffer and boiling for 10 min at 70 °C, resolved by SDS-polyacrylamide gel electrophoresis (PAGE), and analyzed via western blot analysis.

### Western blot analysis

After SDS-PAGE, proteins were transferred to a 0.45 mm polyvinylidene difluoride Immobilon-P membrane (ThermoFisher, IPVH00010) and blocked for 1 h at room temperature (RT) with 5% phosphate-buffered saline-Tween-20 (PBS-T) milk. Membranes were incubated overnight with primary antibodies against the following: LC3 (Novus Biologicals, NB100-2220) 1/3000; β-actin (Abcam, #ab8226) 1/10,000; Map4k3 (Cell Signaling, 9613) 1/1000; RagA (Cell Signaling, 4357) 1/1000; RagC (Cell Signaling, 5466) 1/1000; Lamtor1 (Cell Signaling, 8975) 1/1000; TFEB (Cell Signaling, 4240) 1/1000; pan 14-3-3 (Santa Cruz Biotechnology, sc-629) 1/1000; FLAG (M2) (Sigma; F1804); Raptor (24C12) (Cell Signaling, 2280) 1/1000; phospho-(Ser) 14-3-3 Binding Motif (Cell Signaling, 9601) (for detection of TFEB phosphorylation at Ser211) 1/1000; GST (Santa Cruz Biotechnology, sc-138) 1/1000, in 5% bovine serum albumin in PBS-T. Species-specific secondary antibodies were goat anti-rabbit IgG-HRP (Santa Cruz, sc-2004) or goat anti-mouse IgG-HRP (Santa Cruz, sc-2005), diluted 1/10,000 in 5% PBS-T milk and incubated for 1 h at RT. Chemiluminescent signal detection was captured with Pierce ECL Plus Western Blotting Substrate (ThermoFisher, 321-32) and autoradiography film, using standard techniques. Densitometry analysis was performed using ImageJ. All uncropped scans of immunoblots may be viewed in Supplementary Fig. 9.

### RNA analysis

RNA was extracted from cells using Trizol (ThermoFisher, 15596026). For quantification of messenger RNA expression, complementary DNA synthesis was performed using SuperScript III first-strand synthesis system (ThermoFisher, 18080051) and quantitative reverse-transcriptase PCR (qRT-PCR) was performed using SyberGreen master mix (ThermoFisher, 4367659) and probes^30^. qRT-PCR samples were prepared in triplicate and fold change determined after normalization to GAPDH and negative control treatment conditions. All quantitative PCR was performed on an Applied Biosystem HT7500 Real-Time PCR machine.

### Immunocytochemistry

Cells were seeded in CC2-coated eight-chamber slides (ThermoFisher, 154941) 2 days before experimentation and transfected as indicated. PBS-MC (1 mM MgCl_2_, 0.1 mM CaCl_2_, in PBS) was used for all washes and as a diluent for all solutions. Cells were fixed with 4% paraformaldehyde in PBS-MC for 12 min, then washed three times. Then, 0.05% (HEK293A cells) or 0.2% (HeLa cells) Triton-X in PBS-MC was used to permeabilize the cells for 5 min, followed by two washes in PBS-MC. Primary antibodies used were as follows: TFEB (Cell Signaling, 4240) 1/50, LC3 (MBL, PMO36) 1/3000, FLAG (M2) (Sigma, F1804) 1/2000, Myc (gift from Dr Tony Hunter) 1/7000, LAMP-2 (Santa Cruz, sc-18822) 1/500, and MAP4K3 (Cell Signaling, 92427) 1/500, all diluted in 5% normal goat serum in PBS-MC. Cells were incubated in primary antibodies for 2 h at RT, followed by four washes in PBS-MC. Cells were incubated in secondary antibodies (Alexa Fluor, ThermoFisher) in 5% normal goat serum in PBS-MC for 1 h at RT. Cells were washed four times in PBS-MC, then mounted with Prolong gold antifade reagent with DAPI (ThermoFisher, P-36931) or Hoechst. Images were captured with a Zeiss LSM 780 confocal microscopy or Zeiss LSM 880 airy scan, and analyzed with Zen 2011 LSM 780 software (Salk Biophotonics Core Facility) and Image J.

### Autophagy assays

For autophagic flux determination based on LC3 immunoblotting, we measured LC3-II and actin levels by densitometry using NIH ImageJ and divided LC3-II:actin in the presence of NH4Cl by LC3-II:actin at baseline. To assay autophagic flux in HEK293A and HeLa cells transfected with the mCherry-EGFP-LC3 vector (Addgene, 22418), we performed confocal imaging as above, categorized puncta as yellow (autophagosomes) or red (autolysosomes), imaged ≥ 50 cells per experimental condition from at least three fields per transfection, and performed at least three separate experiments per condition.

### In vitro kinase assay and phosphopeptide mapping

Respective proteins were transfected in HEK293A cells using Lipofectamine 2000 and immunoprecipitated individually, using Dynabeads. Immunoprecipitated TFEB protein was treated with 500 uM 5′-(4-Fluorosulfonylbenzoyl)adenosine hydrochloride for 15 min at 37 °C to inhibit potential kinases that may have co-immunoprecipitated with TFEB. TFEB immunoprecipitates were then washed thoroughly with kinase buffer (25 mM Tris/HCl pH 7.5, 10 mM MgCl_2_) before being combined into new tubes with MAP4K3 immunoprecipitates, 10 µCi of γ-32P-ATP per reaction, and 500 nM Torin1 in kinase buffer, and incubated at 30 °C for 15 min with constant gentle mixing. Proteins in the kinase reactions were then separated by SDS/PAGE and the gel dried. ^32^P incorporation into TFEB was determined by autoradiography and analyzed with a phosphorimager. For phosphopeptide mapping, ^32^P-labeled TFEB was extracted from the dried gel and precipitated with TCA. The precipitated protein was oxidized, digested with trypsin, lyophilized, and the tryptic peptide mix spotted onto a TLC plate. The peptides were then resolved by electrophoresis and chromatography in two dimensions and visualized by autoradiography. For Phospho-amino acid analysis, 50 cpm of purified phosphopeptide was hydrolyzed by incubation for 60 min at 110 °C in 30 µl of 6 N HCl. The sample was then mixed with stainable standards and resolved in two dimensions by electrophoresis. The phospho-amino acid composition was determined by matching the resultant spots on the autoradiograph with the ninhydrin-stained standards on the cellulose plate.

### Growth assay

Dox-inducible WT and S3A TFEB HeLa cells were induced for 30 h, then seeded at 50,000 cells per well, 25,000 cells per well, or 12,500 cells per well in 24-well plates for evaluation of cell growth at 24, 48, or 72 h, respectively, post seeding. Cell number was evaluated using the Cell Count and Viability Assay on the Nucleocounter NC-3000 (Chemometic). Cell growth was calculated by determining the fold change of cell number at seeding to time analysis.

### Live-cell imaging with confocal microscopy

To characterize MAP4K3 localization, spinning disk confocal microscopy was performed with a Zeiss CSU spinning disk system equipped with an inverted × 40 Plan Apo, numerical aperture 1.3 objective, a Yokogawa spinning disk, Evolve EM-CCD camera, and stage-top incubation system. Illumination was achieved using 488 nm and/or 561 nm laser lines. Post-acquisition images and videos were processed and analyzed using FIJI software. All microscopy was performed at the Waitt Advanced Biophotonics Core (Salk Institute, La Jolla, CA).

HEK293A, RPE, or HEK293T cells were seeded at 50% confluence on Lab-TekII chamber slides mounted on 1.5 German borosilicate cover-glass system for 24 h. We obtained an expression vector containing mNeon-Green from Allele Biotech^47^. A total of 100–500 ng MAP4K3-mNeonGreen plasmid was transfected into cells using Lipofectamine 2000 according to the manufacturer’s protocol and incubated in DMEM with 10% FBS overnight at 37 °C. To visualize acidic compartments, LysoTracker Red DND-99 was added to cellular media per manufacturer’s protocol at 37 °C in amino acid-depleted media or amino acid-rich media. Cells were then washed with fresh medium and replenished with respective media. During live-cell imaging, cells were maintained at 37 **°**C and 5% CO_2_ at all times. To image amino acid-dependent effects on MAP4K3 dynamics, cells were either starved of amino acids for at least 1 h before imaging or visualized during amino acid starvation. Cells were then imaged for different time lengths and MAP4K3-mNeonGreen-expressing cells were replenished with amino acids for 10 min. All images were acquired using Zen software (Zeiss).

### Lysosomal fractionation

Lysosomes were isolated from cultured HEK293A cells after disruption of the plasma membrane by dounce homogenizer and sequential centrifugation in a discontinuous Nycodenz density gradient. In short, cells were collected, resuspended in 0.25 M sucrose, and then homogenized until 50% of the cells were disrupted. The homogenate was centrifuged at 6800 *g* for 5 min, and then the resulting supernatant was centrifuged at 17,000 *g* for 10 min. The pellets were resuspended with 0.25 M sucrose until a final volume of 1.1 mL and then mixed well with 2.2 mL 85.6% Nycodenz. The prepared samples were loaded on the bottom of an ultra-clear tube and the gradients in the order of 2 mL 32.8% Nycodenz, 3.3 mL 26.3% Nycodenz, and 3.6 mL 19.8% Nycodenz were added. The prepared tubes were centrifuged at 141,000 × *g* for 1.15 h in a SW41 rotor. Lysosome-enriched fractions were collected from the top two layers (P1 and P2) and the isolated fractions were washed by adding 10 volumes of 0.25 M sucrose and centrifuging at 37,000 × *g* for 15 min. The pellets were collected by resuspending in 0.25 M sucrose and the isolated material were analyzed by immunoblotting or hexosaminidase assay.

### Statistical analyses

All data were prepared for analysis with standard spread sheet software (Microsoft Excel). Statistical analysis was done using Microsoft Excel, Prism 5.0 (Graph Pad), or the VassarStats website (http://faculty.vassar.edu/lowry/VassarStats.html). For analysis of variance, if statistical significance (*P* < 0.05) was achieved, we performed post-hoc analysis to account for multiple comparisons. The level of significance (*α*) was always set at 0.05.

### Data availability

The authors declare that all data supporting the findings of this study are available within the article and its Supplementary Information files, or from the corresponding author upon request.

## Electronic supplementary material

Supplementary Information
