# Supplementary material for: MAP4K3 mediates amino acid-dependent regulation of autophagy via phosphorylation of TFEB
Source: Nat Commun. 2018 Mar 5;9:942. doi: 10.1038/s41467-018-03340-7 (PMC5838220; doi:10.1038/s41467-018-03340-7)
Supplement: Supplementary file 1 — Supplementary Information [file 41467_2018_3340_MOESM1_ESM.doc]

**SUPPLEMENTARY FIGURES**

**Supplementary Figure 1. Validation of MAP4K3 knock-out (k.o.) cell lines permit determination of MAP4K3 regulation of endogenous TFEB subcellular localization**

**(a)** Wild-type (WT) and HEK293A cells gene-edited with either of two different sgRNAs (M1 and M4) were lysed, and protein lysates were immunoblotted for MAP4K3. Immunoblotting of β-actin served as a loading control.

**(b)** Knock-out of MAP4K3 yields TFEB nuclear localization. WT HEK293A cells and MAP4K3 k.o. cells were cultured in complete media (CM), or starved of amino acids for 120 min, and then restimulated with amino acids for 10 min. Here we see representative images of cells immunostained with an antibody directed against endogenous TFEB. All experiments were performed in triplicate. Scale bar = 10 μm

**Supplementary Figure 2. Validation of TFEB dependence and constitutively active Rheb construct**

**(a)** TFEB is required for induction of lysosomal gene expression in MAP4K3-depleted cells.

WT HeLa cells or TFEB k.o. cells were transfected with a control shRNA or a MAP4K3 shRNA, and cultured in complete media. Quantitative RT-PCR of isolated RNAs for these cell lines was performed for five TFEB target genes. One-way ANOVA with post-hoc Tukey test; **P* < .05, ***P* < .01. All experiments were performed in triplicate. Error bars = s.e.m.

**(b)** Constitutively active Rheb yields mTORC1 activation in amino acid depleted cells.

WT HEK293A cells or MAP4K3 k.o. cells were transfected with a TFEB expression construct, with or without constitutively active Rheb, as indicated, and subjected to amino acid starvation for 2 hrs. Immunoblotting of cell lysates was performed for the indicated phosphorylated targets of mTORC1, with β-actin serving as a loading control. All experiments were performed in triplicate.

**Supplementary Figure 3. MAP4K3 regulation of TFEB is independent of mTORC1 activation status**

(**a**) Activation of mTORC1 does not alter TFEB localization in MAP4K3 k.o. cells. WT HEK293A cells and MAP4K3 k.o. cells were transfected with an expression construct for constitutively active Rheb, epitope-tagged with myc, and maintained in nutrient replete complete media. Both untransfected WT HEK293A cells (orange arrows) and WT HEK293A cells expressing constitutively active Rheb (white arrows) display TFEB cytosolic localization. MAP4K3 k.o. cells, regardless of whether untransfected (orange arrows), or expressing constitutively active Rheb (white arrows) show that TFEB localizes to the nucleus.

Scale bar = 10 μm

(**b**) Quantification of WT-HEK293A cells and MAP4K3 k.o. cells with predominantly TFEB nuclear localization, under different culture conditions and in the presence or absence of constitutively active Rheb (CA Rheb) expression. n > 50 cells / condition. One-way ANOVA with post-hoc Tukey test; ***P* < .01, ****P* < .001. All experiments were performed in triplicate. Error bars = s.e.m.

**Supplementary Figure 4. Treatment with constitutively active Rheb does not alter TFEB target gene expression in MAP4K3 k.o. cells.**

MAP4K3 k.o. cells were mock-transfected or transfected with constitutively active Rheb, and cultured in CM. Quantitative RT-PCR of isolated RNAs for these cell lines was performed for four TFEB target genes, and no significant differences were found by two-tailed t-test analysis. This experiment was performed in triplicate. Error bars = s.e.m.

**Supplementary Figure 5. MAP4K3 phosphorylates TFEB at serine 3**

WT HEK293A cells were transfected with WT-MAP4K3-FLAG or KD-MAP4K3-FLAG, and either TFEB-FLAG, TFEB-S3A-FLAG, or TFEB-S211A-FLAG, as indicated. FLAG immunoprecipitates were subjected to in vitro kinase reactions with γ-P^32^-ATP, with Torin1 and the general kinase inhibitor FSBA included in the reaction mixture. Phosphopeptide mapping was performed after enzymatic digestion with thermolysin by spotting the resulting peptide mix onto cellulose thin layer chromatography plates, followed by 2D gel electrophoresis and chromatography, and finally autoradiography to visualize phospho-labeled peptides. Circles indicate location of phospho-S3-TFEB. Note absence of phospho-S3-TFEB for TFEB-S3A and for kinase-dead (KD) MAP4K3. This experiment was performed in triplicate.

**Supplementary Figure 6. Cells expressing phosphoresistant TFEB S3A exhibit a reduced growth rate**

(**a**) Validation of TFEB inducible cell lines. We created a TFEB k.o. cell line (TFEB k.o.) and then stably transfected TFEB k.o. cells with either TFEB-WT-FLAG or TFEB-S3A-FLAG. Here we see immunoblotting analysis of the different inducible cell lines in the absence or presence of inducer (doxycycline) in comparison to control HeLa cells and the TFEB k.o. cell line. Note that TFEB migrates at a higher molecular mass in the different inducible cell lines because of the presence of a FLAG epitope tag. Immunoblotting of β-actin served as a loading control.

(**b**) TFEB-S3A expressing cells grow more slowly than TFEB-WT expressing cells. TFEB-WT or TFEB-S3A inducible cell lines were cultured in the absence or presence of doxycycline for 30 hrs before plating, and then tested for cell doubling at 24, 48, and 72 hrs after plating. Growth rate was determined by calculating the fold change in growth of a particular cell line at 24, 48, or 72 hrs after plating in comparison to cell number at time zero. One-way ANOVA with post-hoc Tukey test; **P* < .05.

(**c**) Quantification of the fold change in growth rate for the indicated cell lines at 72 hrs after plating. One-way ANOVA with post-hoc Tukey test; **P* < .05. All experiments were performed in triplicate. Error bars = s.e.m.

**Supplementary Figure 7. Validation of lysosomal fractionation protocol.**

**(a)** Sucrose density ultracentrifugation was performed to achieve fractionation of different subcellular organelles, as shown.

**(b)** To validate the success of the lysosomal fractionation, we performed a hexosaminidase assay on the cell homogenate (starting material) and on the resultant P1 and P2 fractions, as hexosaminidases are present in lysosomes. The detection of enormously high levels of hexosaminidase activity in the P1 fraction in comparison to the P2 fraction and cell homogenate confirms that the P1 fraction is highly enriched for lysosomes. This experiment was performed in triplicate.

**Supplementary Figure 8. MAP4K3 cytosolic localization is regulated by amino acid supply.**

**(a)** Primary retinal pigmented epithelial (RPE) cells and HEK293T cells were transfected with MAP4K3-mNeonGreen and subjected to amino acid starvation for 10 min. Scale bars = 10 μm.

**(b)** HEK293A cells were transfected with MAP4K3-mNeonGreen, treated with Lysotracker Red, and starved of amino acids, before being switched to amino acid replete media. Here we see the appearance of the cells at 10 min after the addition of amino acids. Note diffuse cytosolic localization of MAP4K3 and lack of MAP4K3 localization to cytosolic puncta or co-localization with Lysotracker Red. All experiments were performed in triplicate. Scale bar = 10 μm.

**Supplementary Figure 9. Original uncropped scans of immunoblots**

Figure 1a Figure 1b


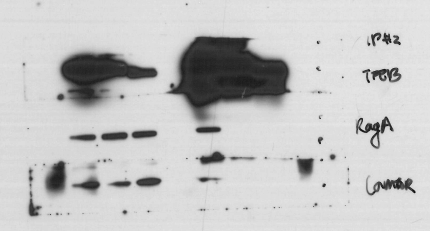


Figure 4a


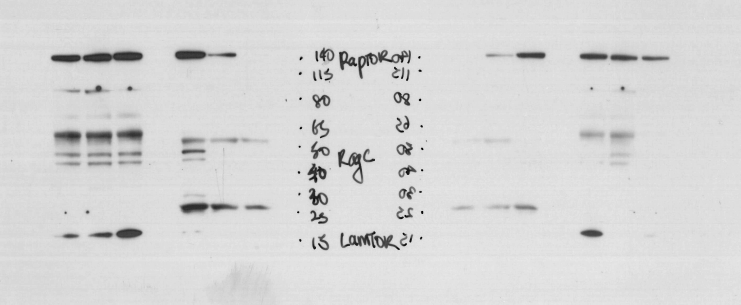


Figure 4a


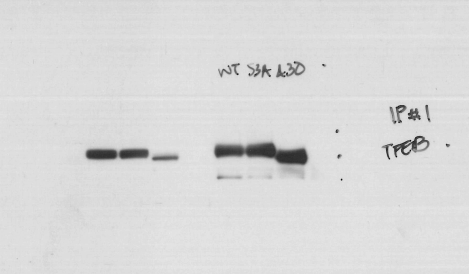


Figure 4a


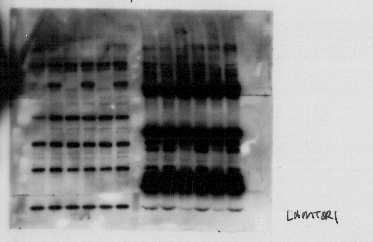


Figure 4b


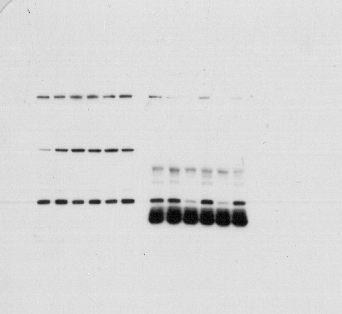

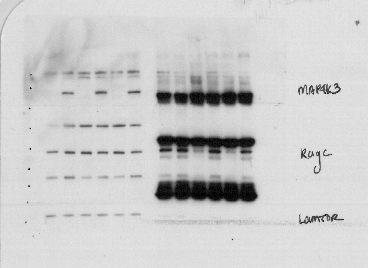


Figure 4b Figure 4b


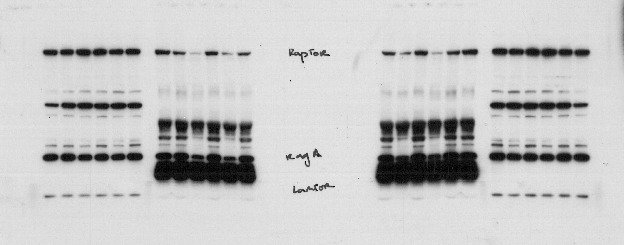


Figure 4b


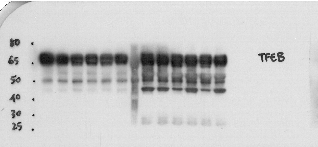


Figure 4b


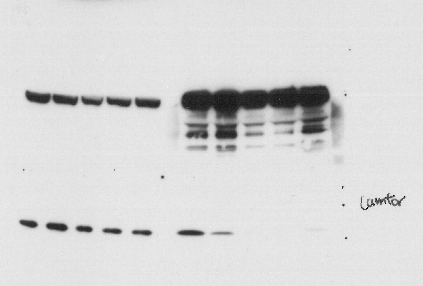

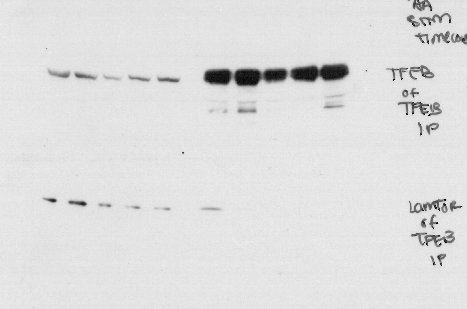


Figure 4c Figure 4c


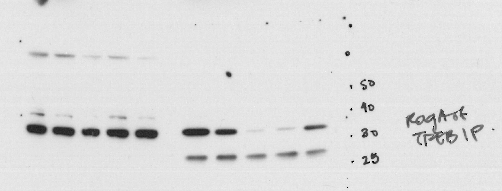


Figure 4c


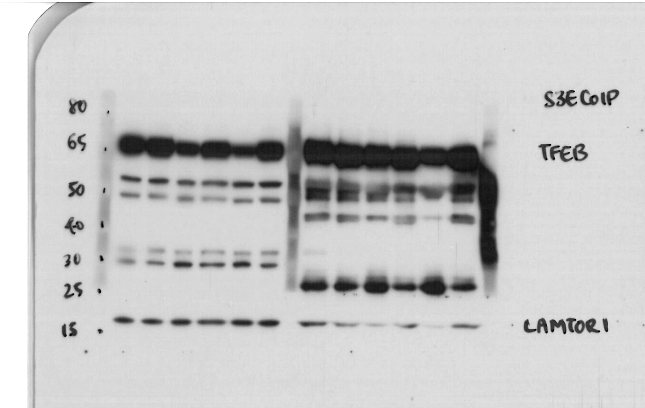


Figure 4d


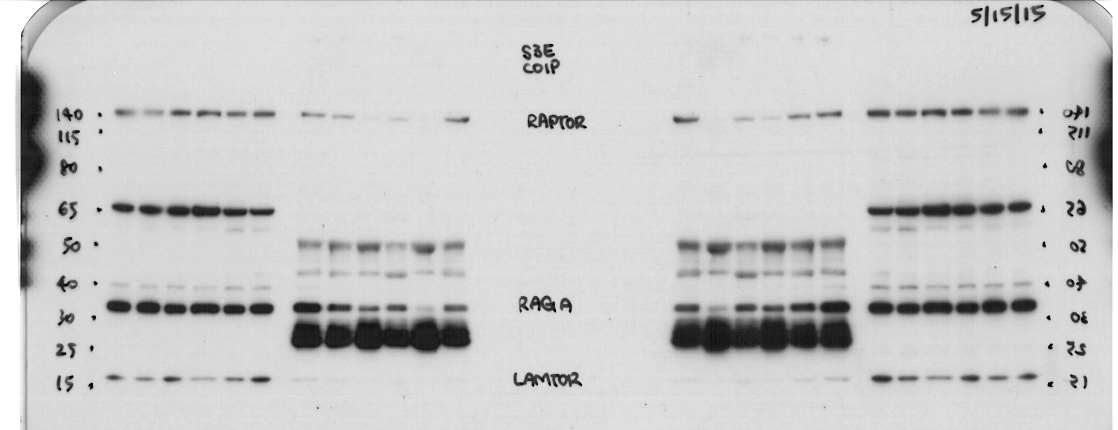


Figure 4d


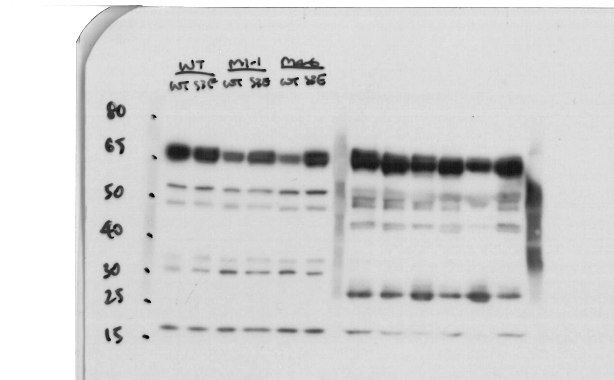


Figure 4d


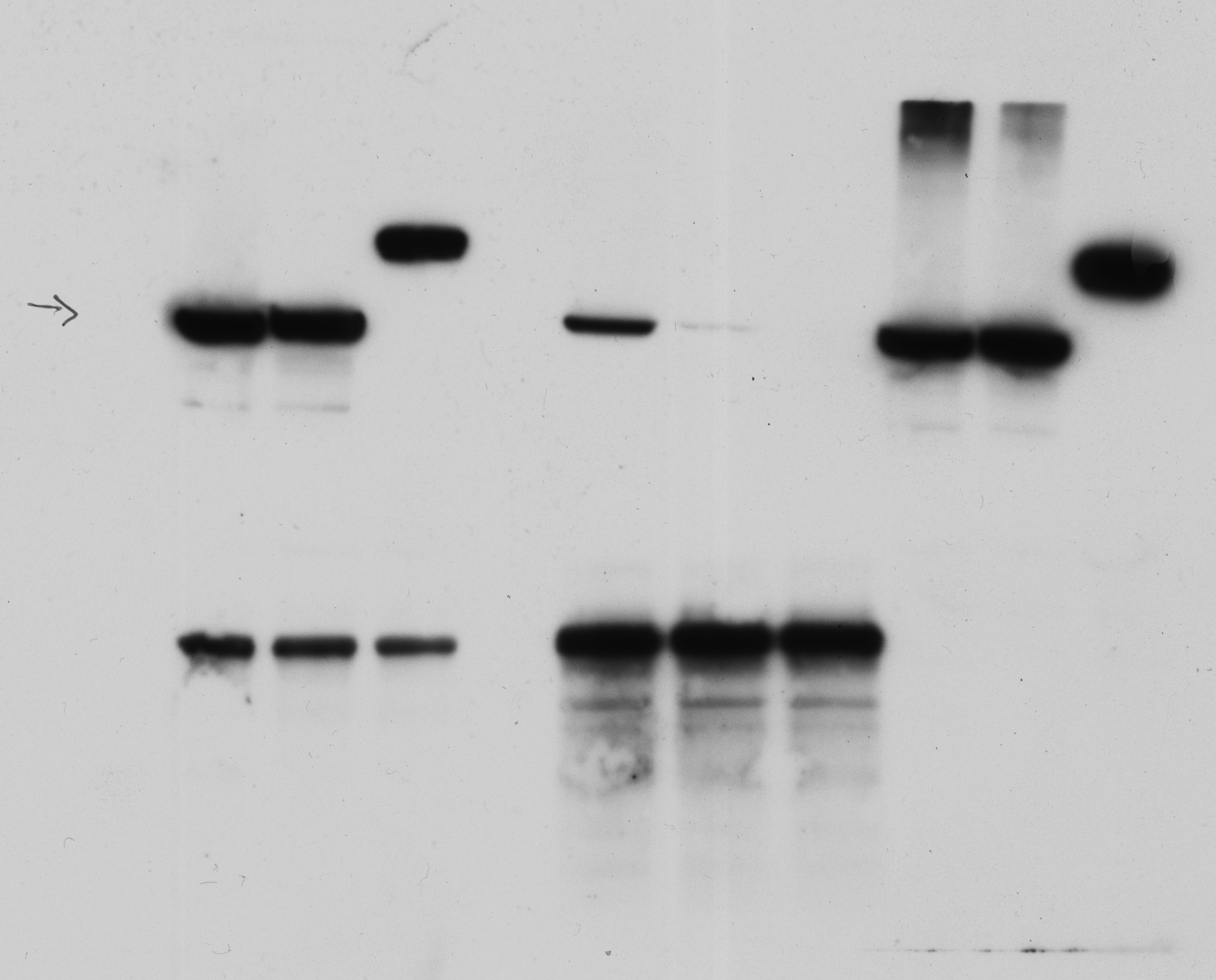

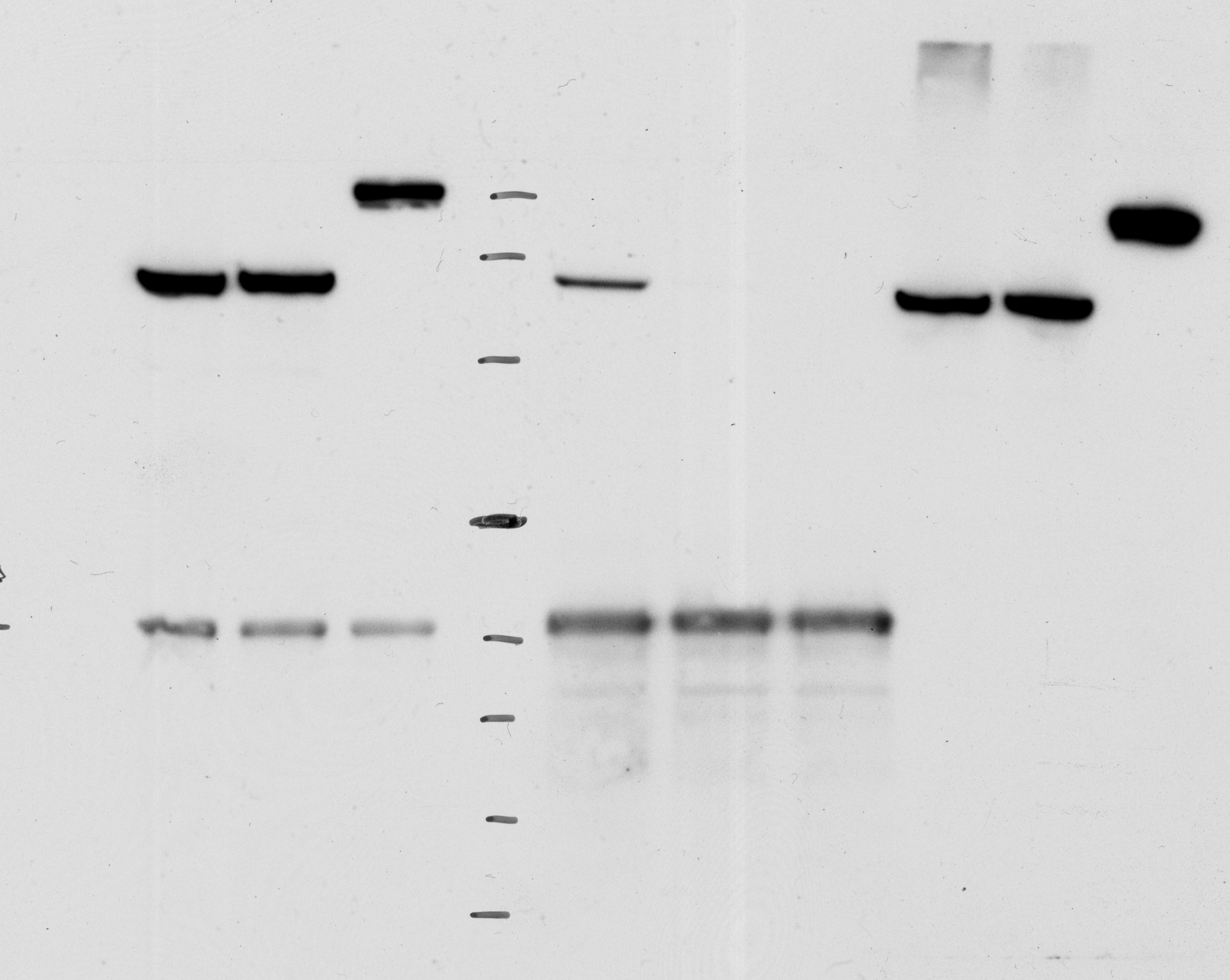


Figure 5a


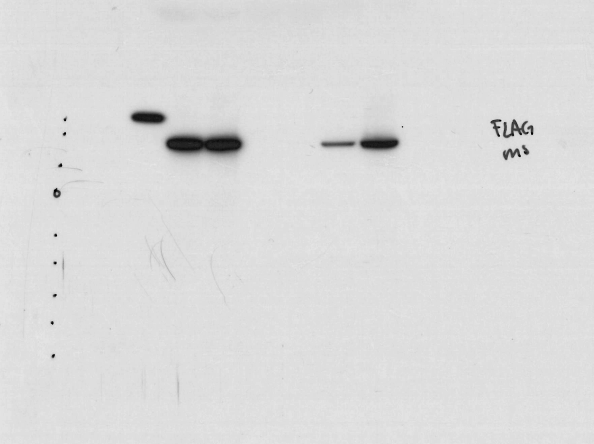

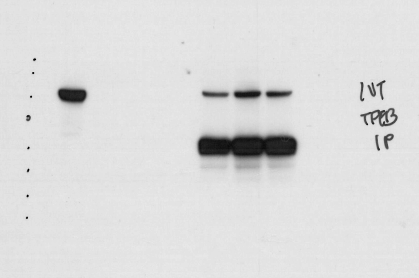


Figure 5b


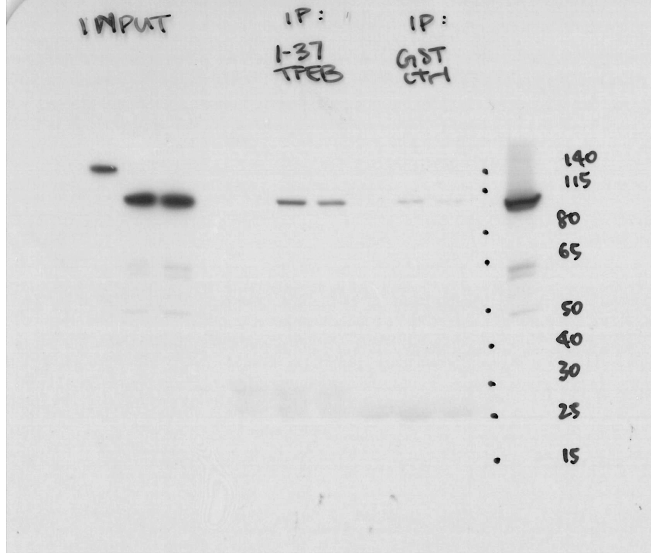

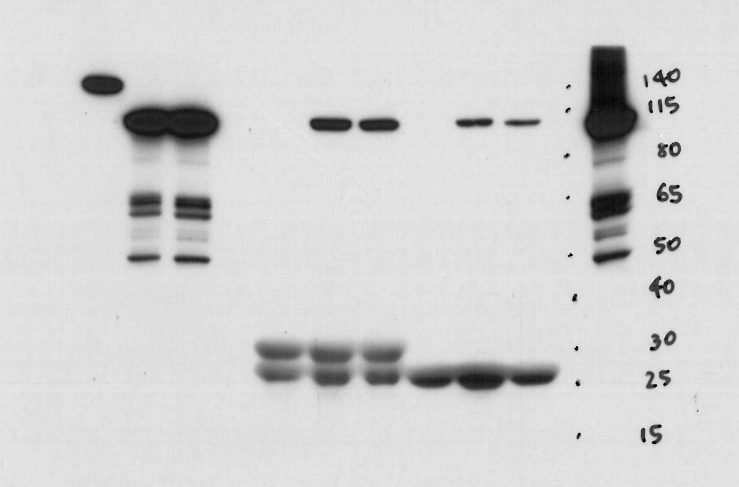


Figure 5c


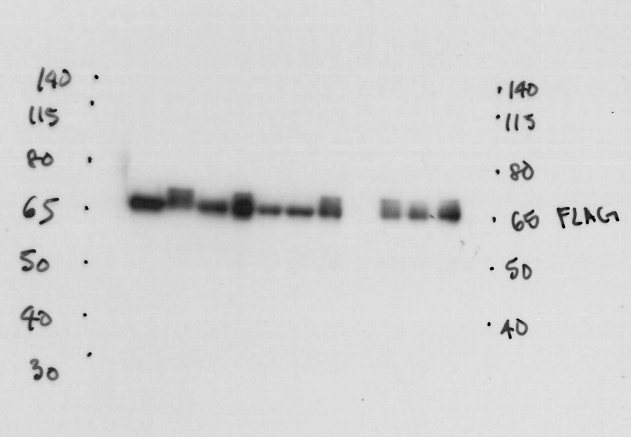

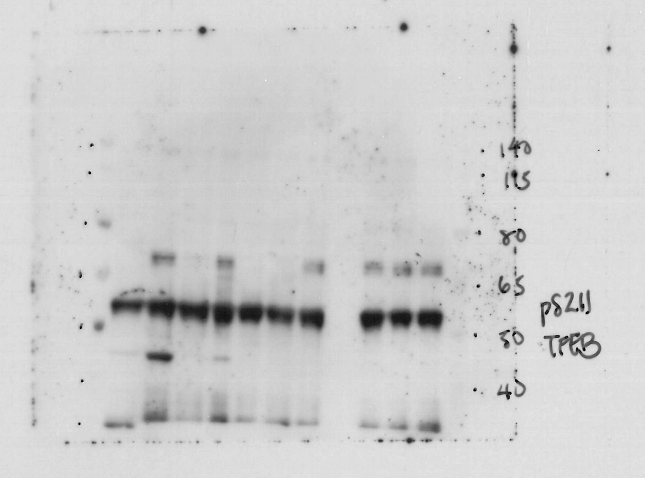


Figure 6a


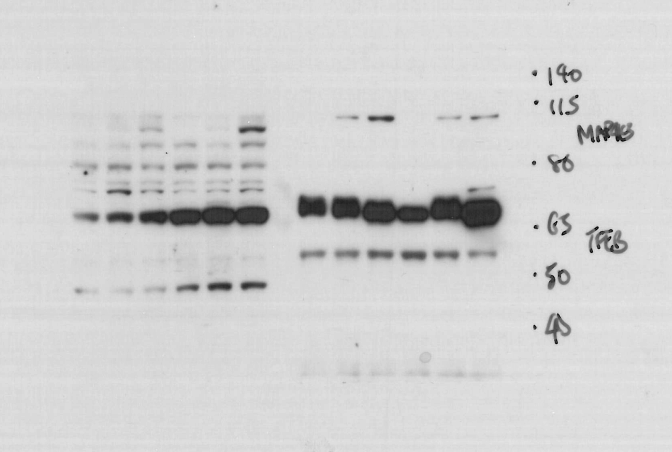

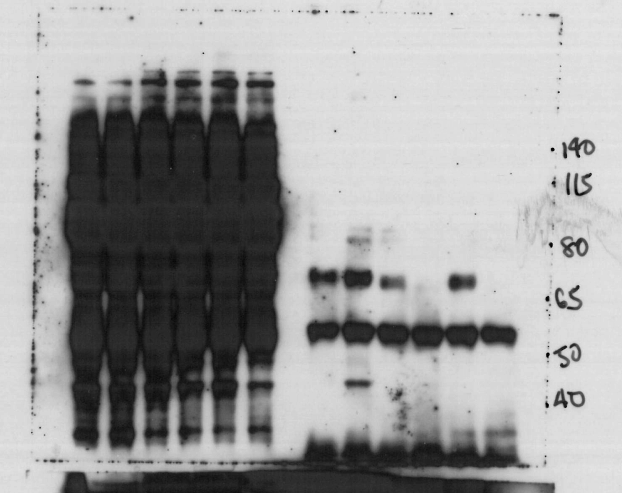


Figure 6b


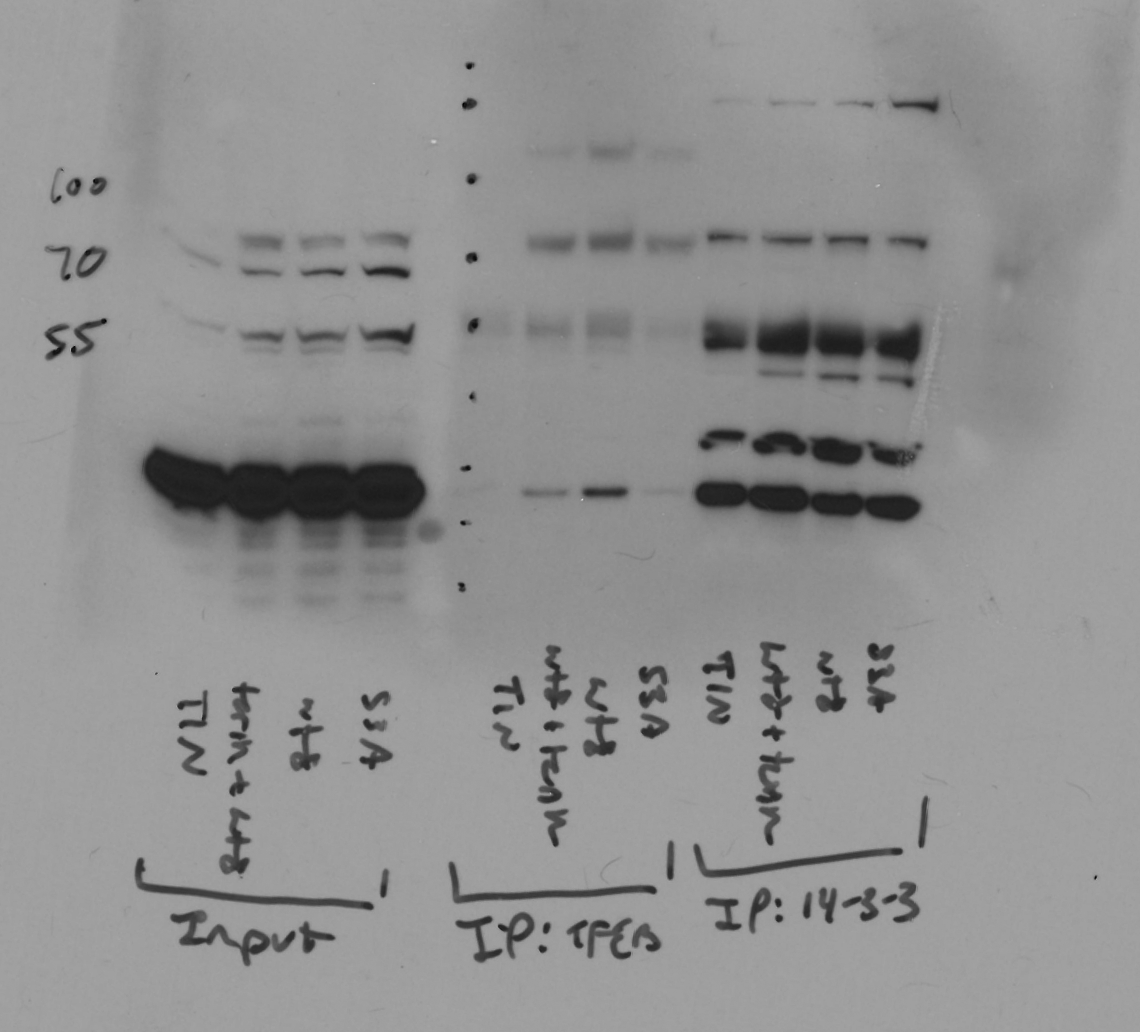

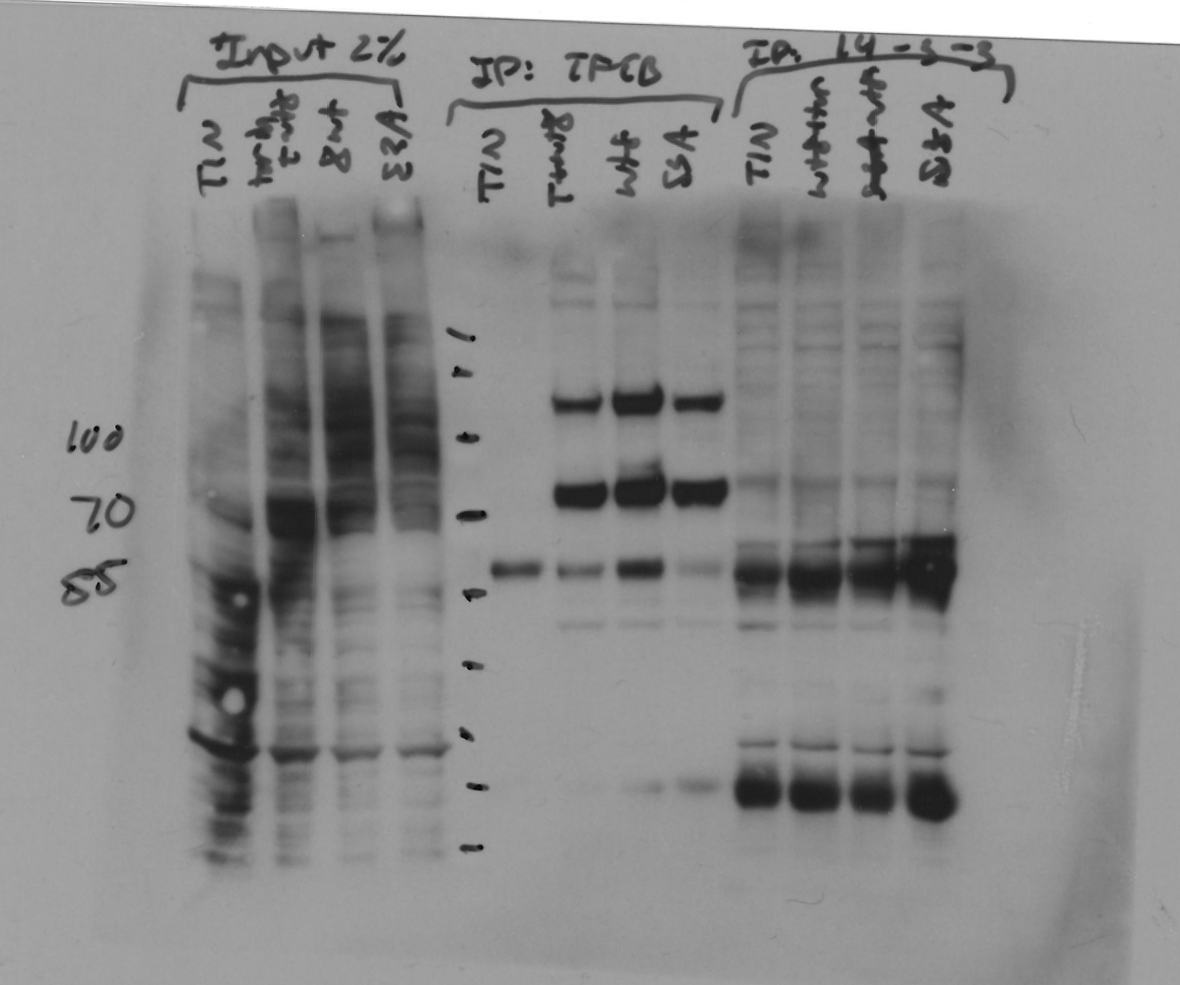


Figure 6c


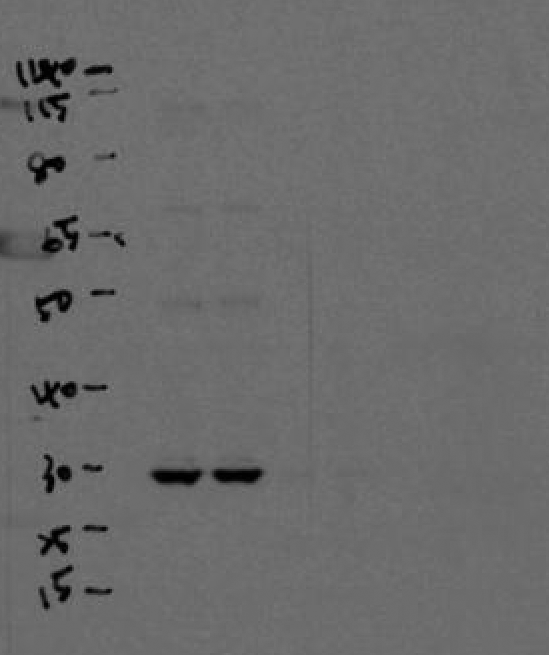

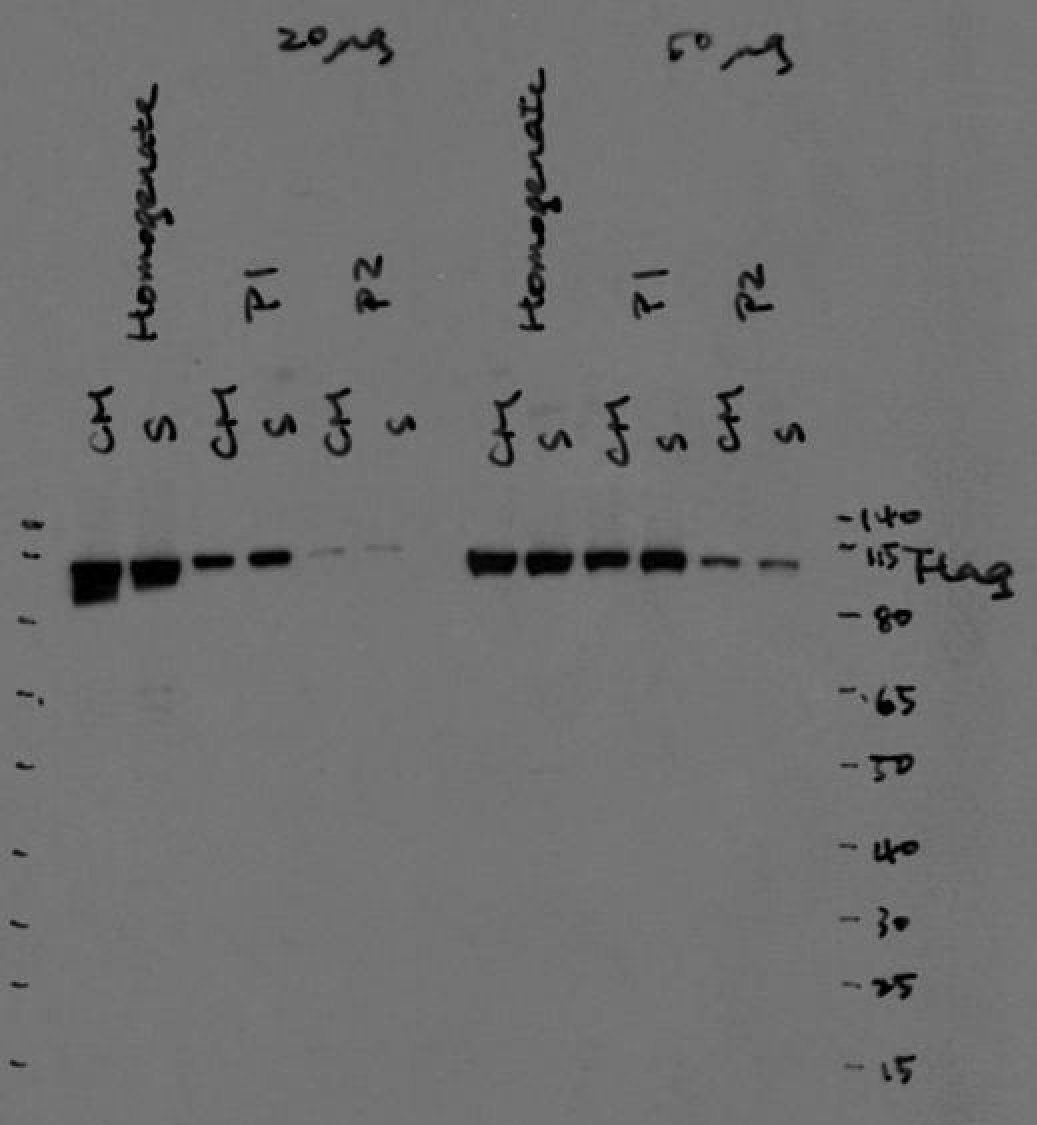

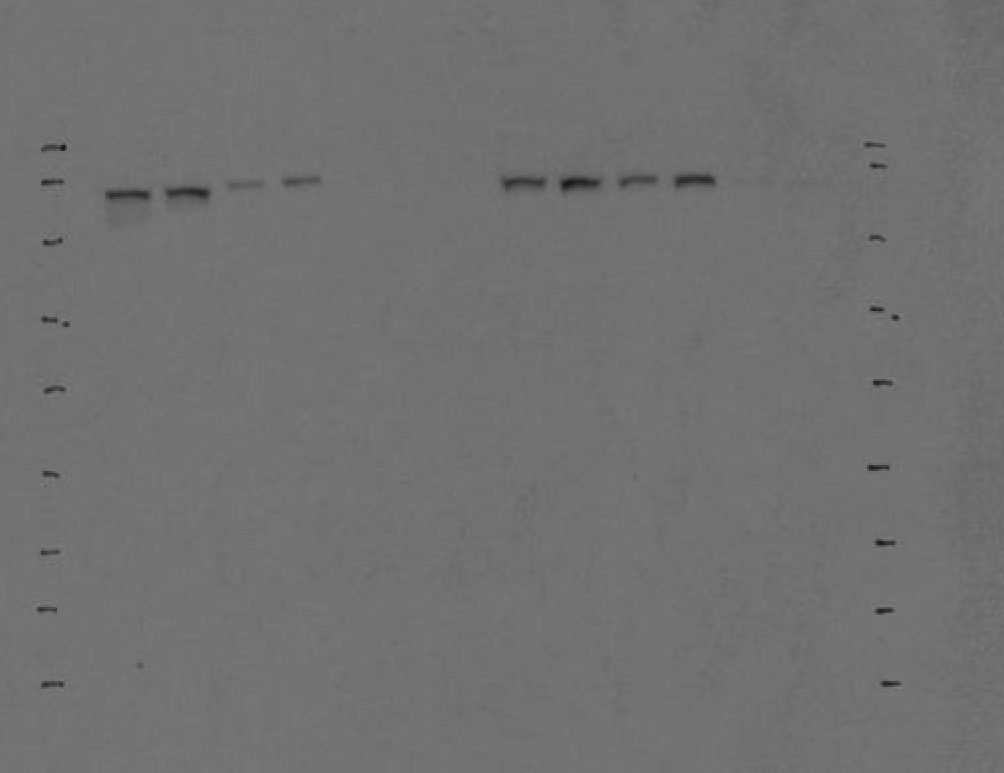

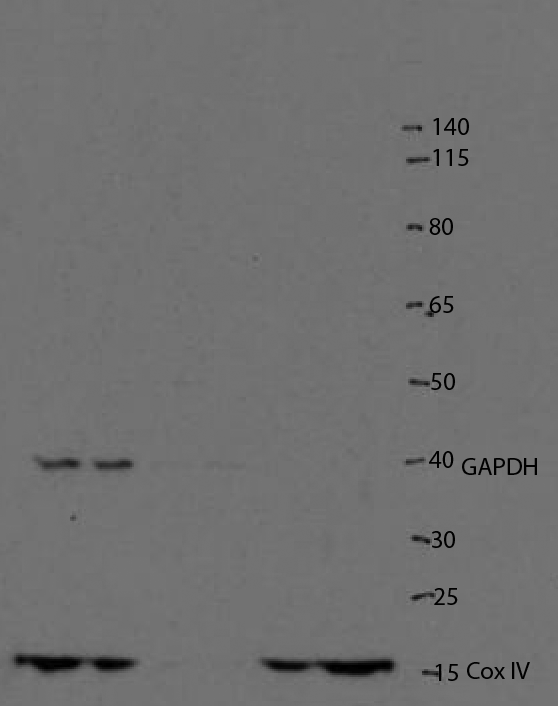

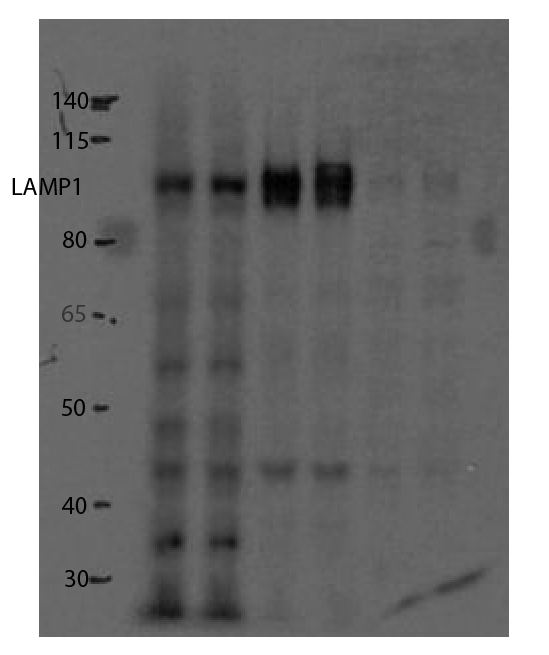


Figure 7a


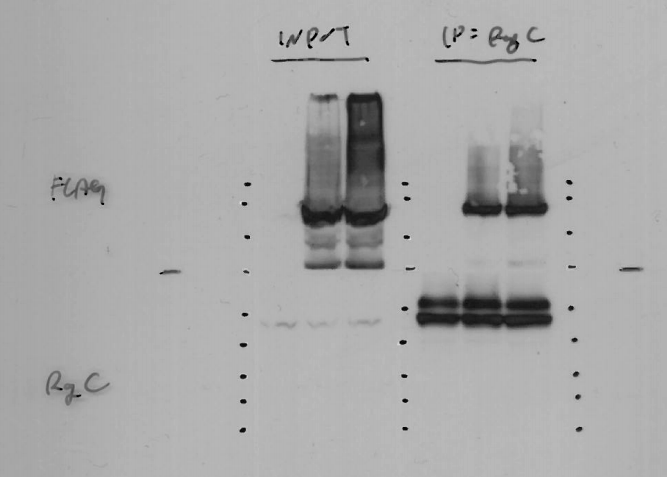

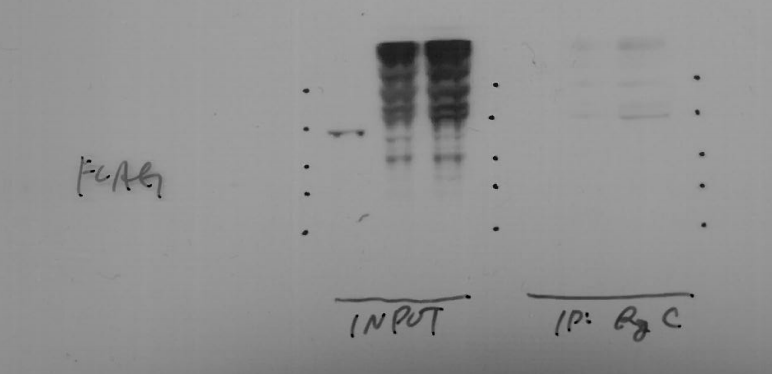


Figure 8c


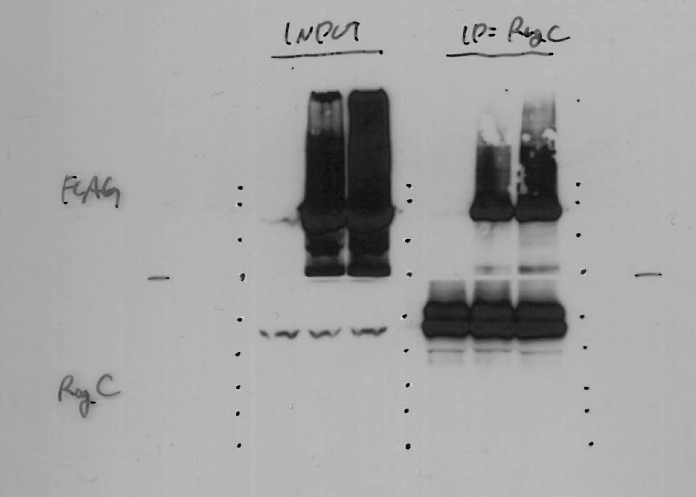


Figure 8c


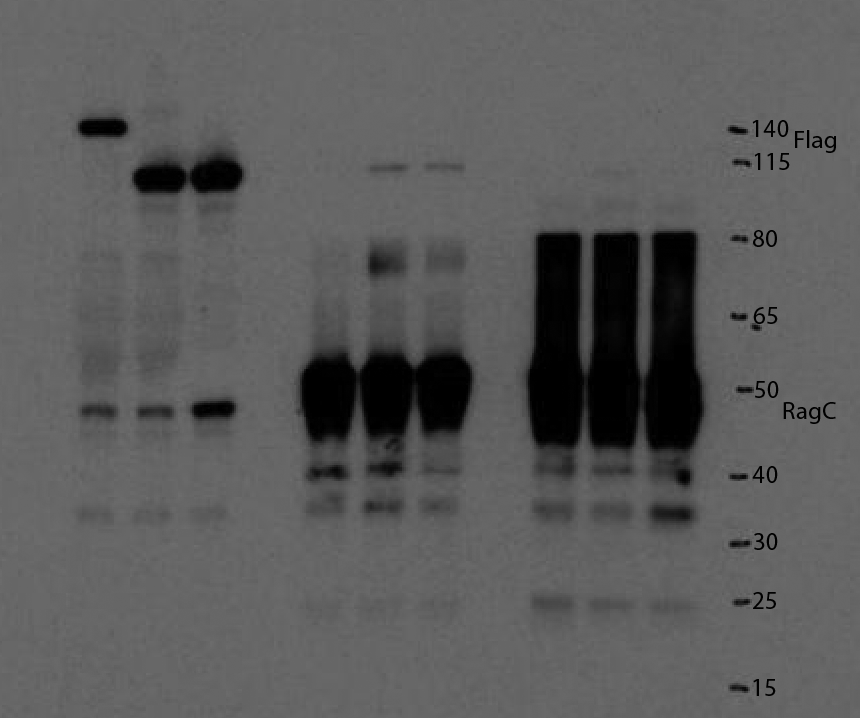


Figure 8d

Figure 8d


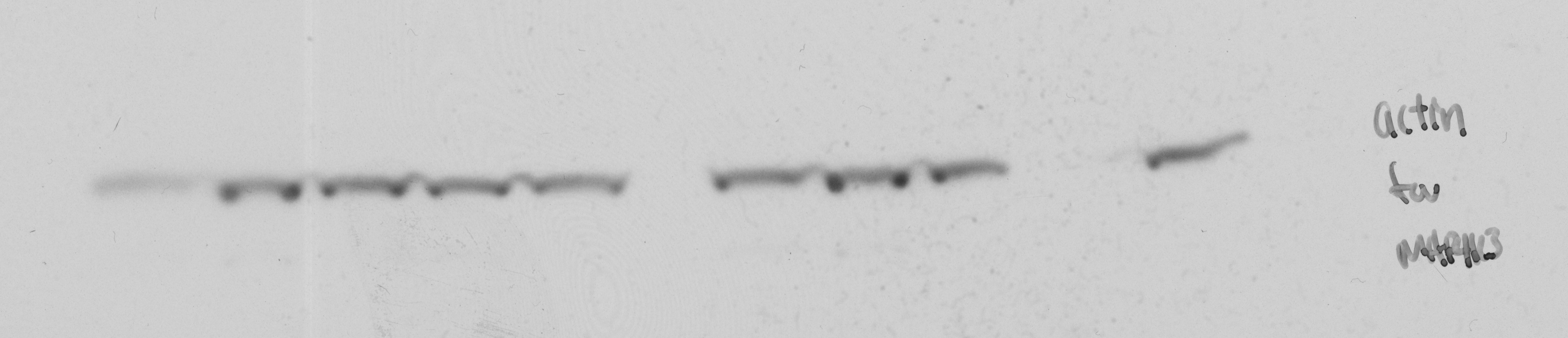

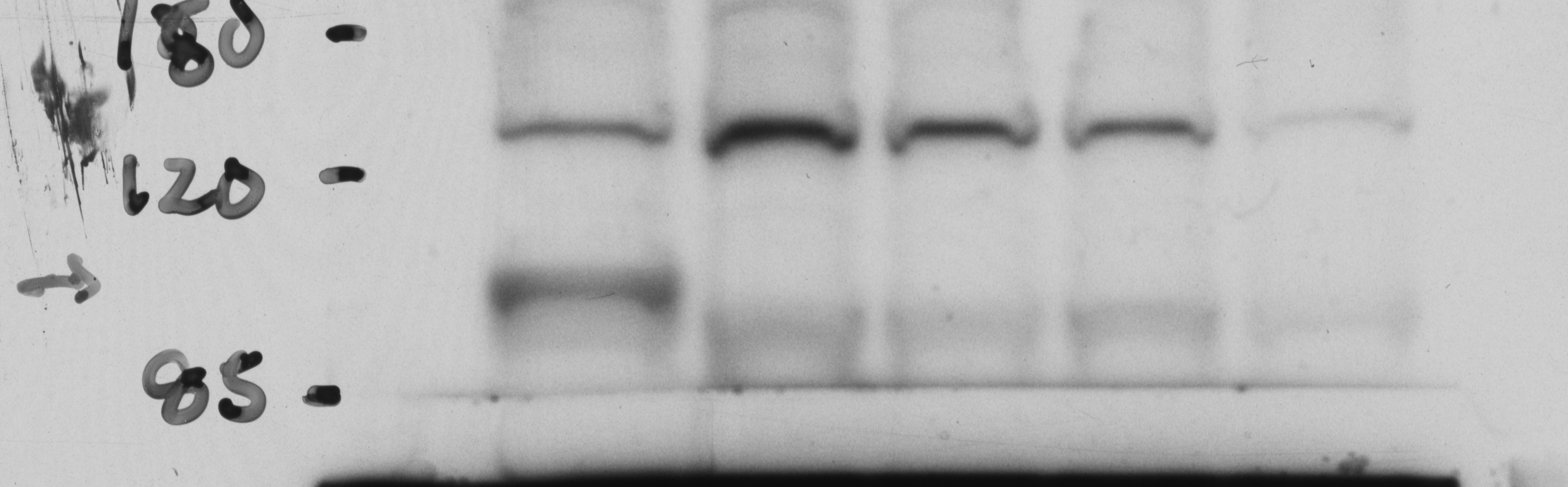


Suppl Figure 1a


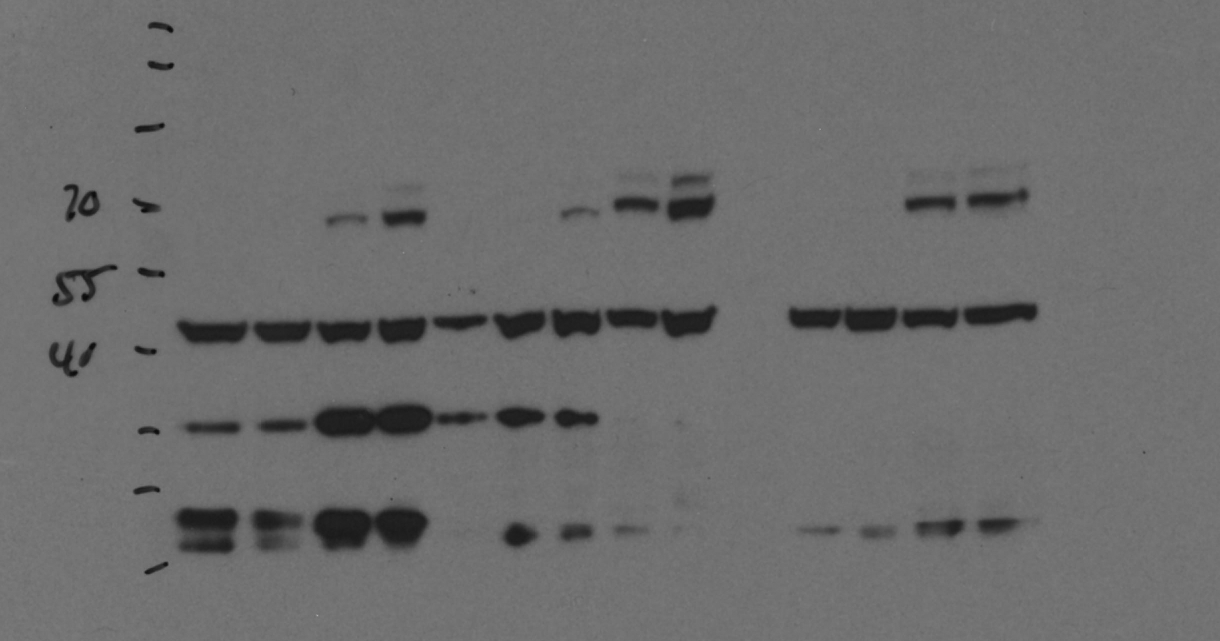


Suppl Figure 2b


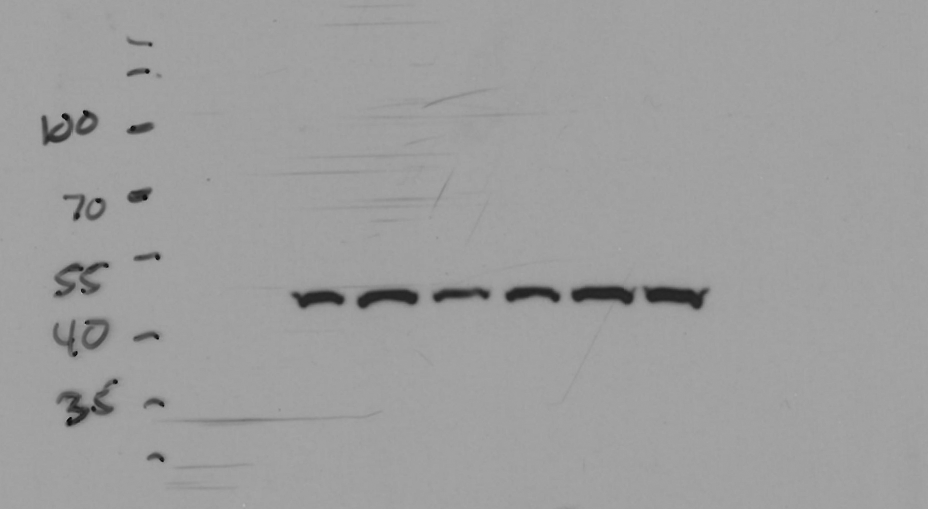

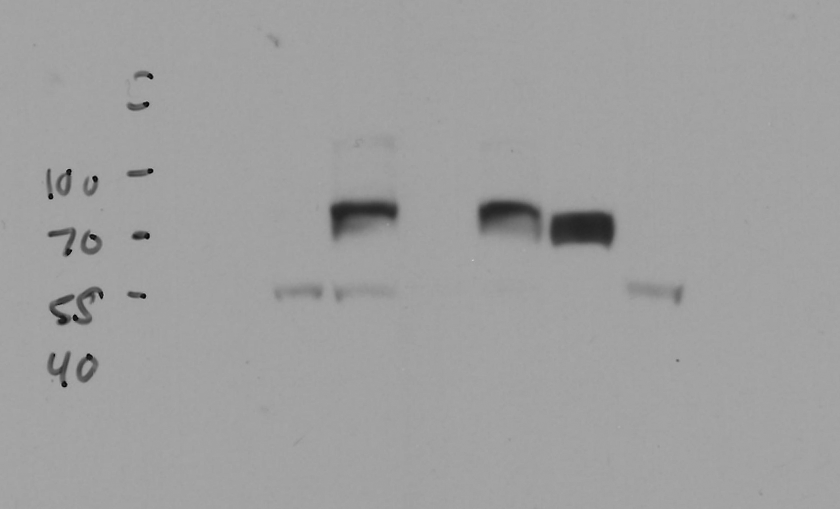


Suppl Figure 6a
